# Inducing Targeted, Caspase-Independent Apoptosis with New Chimeric Proteins for Treatment of Solid Cancers

**DOI:** 10.3390/cancers17071179

**Published:** 2025-03-31

**Authors:** Orly Melloul, Samar Zabit, Michal Lichtenstein, Deborah Duran, Myriam Grunewald, Haya Lorberboum-Galski

**Affiliations:** 1 Department of Biochemistry and Molecular Biology, Faculty of Medicine, Institute for Medical Research Israel-Canada (IMRIC), The Hebrew University of Jerusalem, Jerusalem 9190501, Israel; orly.elbaz@mail.huji.ac.il (O.M.); samar.zabit@mail.huji.ac.il (S.Z.); michallic@ekmd.huji.ac.il (M.L.); 2 Hadassah Organoid Center, Hadassah University Medical Center, Jerusalem 9112102, Israel; deborah.duran@mail.huji.ac.il (D.D.); myriamg@hadassah.org.il (M.G.); 3 Department of Developmental Biology and Cancer Research, Faculty of Medicine, Institute for Medical Research Israel-Canada (IMRIC), The Hebrew University of Jerusalem, Jerusalem 9190501, Israel

**Keywords:** caspase-independent apoptosis, GnRH, GnRH-receptor (GnRH-R), apoptosis-inducing factor (AIF), chimeric proteins, targeted cancer treatment, solid cancers

## Abstract

**Background:** Most newly developed anticancer treatments trigger tumor cell death through apoptosis, for which involvement of caspases activity is essential. However, numerous mutations in apoptotic pathways that lead to cancer and favor resistance to apoptosis are known; most are related to caspase-dependent apoptosis pathways and thus have low efficacy. To overcome these limitations, we constructed a novel chimeric protein, GnRH-AIF, using a gonadotropin-releasing hormone (GnRH) analog as a targeting moiety and the apoptosis-inducing factor (AIF) in its cleaved form as a killing moiety, fused at the cDNA level. AIF has a crucial role in the caspase-independent apoptotic pathway. A wide variety of solid tumors overexpress GnRH-receptors (GnRH-R) that are targeted by the new GnRH-AIF chimeric protein. **Methods and Results:** In this study, we constructed, expressed, and highly purified GnRH-AIF chimeric proteins. We demonstrated the ability of the chimera to enter and specifically and very efficiently kill solid cancer cell lines overexpressing GnRH-R. Most importantly, upon its entry, GnRH-AIFs translocate to the nucleus where it causes DNA fragmentation leading to a direct caspase-independent apoptotic death. As AIFs lack nuclease activity, our findings also emphasize that cell death induced by GnRH-AIF is dependent on the presence of the ENDOG and PPIA proteins, known to participate in the formation of a DNA–degradosome complex. Finally, we demonstrated the high anti-tumor efficacy of the GnRH-AIF ex vivo, in a human, colon cancer organoid model. **Conclusions:** Our study shows the potential of using a GnRH-AIF chimeric protein as a novel approach to treat solid cancers that overexpress GnRH-R, via a caspase-independent apoptotic pathway.

## Simple Summary

Despite major advances in modern medicine, most solid tumors remain sill incurable. These anticancer treatments trigger tumor cell death through programmed cell death (apoptosis), for which involvement of proteases (caspases) activity is essential. However, cancer cells are known to harbor mutations in various components of this classical apoptotic pathway which leads to treatment failure. Thus, in this study we developed a new molecule, a GnRH-AIF chimeric protein for targeted, caspase-independent cell death of cancer cells. The GnRH-AIF chimeric protein is composed of a gonadotropin-releasing hormone (GnRH), targeting a specific cell surface receptor overexpressed on several types of solid tumors, the GnRH-receptor, and a natural human cellular protein, the soluble apoptosis-inducing factor (AIF), which is causing caspase-independent, apoptotic cell death. Our results demonstrated the ability of GnRH-AIF chimeric proteins to target, enter, and kill specifically and efficiently human cancer cells overexpressing GnRH-R by causing caspase-independent, apoptotic death, as an innovative approach to the targeted treatment of solid cancers.

## 1. Introduction

Cancer research over five decades has resulted in the generation of enormous amounts of information and drugs for targeted cancer therapy. Advanced and innovative cancer therapies are the following: stem cell therapy, pluripotent stem cells, and adult stem cells; immunotherapy including monoclonal antibodies, checkpoint inhibitors, CAR-T cell treatment, adoptive cell transfer, and cancer vaccines (therapeutic vaccinations and preventative vaccines); gene therapy including tailored small interfering RNAs (siRNAs) for gene silencing of antiapoptotic proteins, transcription factors, or cancer-related mutated genes and gene editing (CRISPR-Cas9); hormonal therapy; oncolytic virus therapy; molecular targeted therapy for disrupting the growth molecules responsible for inhibiting the development and metastasis of cancer; hyperthermia; and photodynamic therapy and additional treatments [1,2,3,4,5]. However, relatively little progress has been made in the treatment of patients with solid tumors, other than extending their survival for a few months at best. Moreover, after half a century of extensive research and numerous clinical trials, even with novel designed targeted molecules, cancer remains the second-leading cause of death in the United States and, by inference, in other developed countries [1]. Most newly developed anticancer treatments trigger tumor cell death through apoptosis, where involvement of caspases’ activity is essential. However, numerous reviews have reported mutations in apoptosis pathways that lead to cancer and favor cell resistance to apoptosis; most of these are related to caspase-dependent apoptosis pathways [2,3]. These include mutations involved in upstream caspase activation that leads to cancer, such as mutations involving the death receptors (DRs) [4], various caspases [5,6,7], and nucleases [8]. Mutations or dysregulated expression of proteins of the Bcl-2 family proteins have also been widely identified in numerous cancers as well [9,10]. Indeed, tumor cells are often resistant to classical caspase-mediated pathways because of excessive expression of anti-apoptotic proteins, such as Bcl-2. Due to these limitations, it is becoming essential to identify and design novel, effective targeted anti-tumor agents that induce caspase-independent programmed cell death. In this study, we aimed to design a new molecule that will induce caspase-independent cell death for the treatment of solid tumors in the form of a chimeric protein.

Chimeric proteins, designed and constructed by gene fusion techniques, comprise both the cell targeting and the active moieties and belong to the “toxin delivery molecules” category of targeted cancer therapies. The first generation of these molecules, termed immunotoxins, consisted of bacterial or plant toxins as the active moiety of the chimeras that were conjugated or fused to an antibody [11]. An example of an immunotoxin with FDA approval is moxetumomab pasudotox-tdfk (Lumoxiti™), an anti CD22 Pseudomonas exotoxin A (PE) recombinant immunotoxin, for the treatment of hairy cell leukemia [12]. In this regard, a chimeric protein for the treatment of solid cancer cells was previously constructed by us with an analog of the decapeptide gonadotropin-releasing hormone (GnRH) serving as the targeting domain, fused to the full-length modified bacterial PE. GnRH-PE chimeric proteins had the ability to bind, internalize, and kill a large number of human-originated cancer cell lines as well as having an anti-tumor effect in vivo in a human colon xenograft mouse model [13].

Our targeting moiety in these chimeric proteins was GnRH, which is normally synthetized by the hypothalamic neurons and secreted in a pulsatile manner [14]. Upon reaching the anterior pituitary gland, GnRH binds to specific receptors and activates the GnRH receptor (GnRH-R). The result of its binding is the selective stimulation of the gonadotrophin cells to release luteinizing hormone and follicle-stimulating hormone, thus playing a central role in the control of human reproduction [14]. GnRH-R belongs to the 7-transmembrane G protein coupled receptor family [14]. There are two types of GnRH-R, type 1 and type 2. Type 1 is involved almost exclusively in the reproductive system whereas type 2 is expressed throughout the brain and not confined to the pituitary [14]. However, in humans, the N-terminus of the predicted protein GnRHR2 contains a frameshift and premature stop codon. In humans, GnRHR2 transcription occurs, but whether the gene produces a functional protein is currently unresolved [14]. It has become increasingly clear that GnRH-R1s are overexpressed in cancer tissues, either related (prostate, breast, endometrial, and ovarian cancers) or unrelated (colon, melanoma, glioblastoma, lung, pancreatic cancers, and more) to the reproductive system [15]. We were the first to report that a wide variety of solid tumors, not connected to the reproduction system, i.e., non-hormonal originated cancers, overexpress the specific GnRH-R [16]. In the last few years, the use of GnRH analogs has been proposed for treatment of various hormone-dependent cancers, via inhibition of estrogen and androgen synthesis, and today are used as an androgen-deprivation treatment of prostate cancer [17].

The overexpression pattern of GnRH-R makes it an attractive target to deliver therapeutic agents exclusively to tumor cells. Although GnRH-R is expressed in the pituitary gland, it has been reported that GnRH-targeted proteins were not able to escape through the barriers that separate the pituitary gland from the systemic circulation so had no effect on normal pituitary function [18]. Therefore, the GnRH-GnRH-R system can be considered as an efficient system to target a variety of solid cancer cells. Indeed, in recent years we have developed new chimeric proteins introducing human apoptosis-inducing proteins of the Bcl2 family as well as a specific apoptotic nuclease, instead of the bacterial toxins, to reduce immunogenicity and off-target problems. These chimeric proteins, GnRH-Bik, GnRH-Bak, GnRH-Bax, and GnRH-DFF40, efficiently and specifically inhibited the cell growth of human cancer cell lines [19]. Recently, similar chimeric proteins were also developed by others with significant positive results [20]. However, all these chimeric proteins induce the caspase-dependent apoptotic death of cancer cells both in vitro and in vivo in cancer models in mice. In the current study, we developed and tested new GnRH-based chimeric proteins that induce caspase-independent apoptotic death for treatment of solid cancers.

Searching for human proteins known to induce caspase-independent apoptotic death, we examined the apoptosis-inducing factor (AIF) protein, as a new “killing moiety” for our targeted chimeric proteins. AIF is a flavoprotein that is synthesized in the cytosol and imported into the mitochondrial Inner Membrane (IMS). The AIF precursor carries a mitochondrial localization sequence (MLS) in its N-terminal pre-sequence. Under normal conditions, the 54-residue MLS is first proteolytically removed after translocation into the mitochondrial intermembrane space to form the mature form (AIF-mit) where it has a NADH-dependent oxidoreductase activity [21,22]. During apoptosis, it is further proteolytically processed at amino-acid position 101, leading to the generation of the soluble form (AIF-sol). AIF-sol is released to the cytoplasm in response to specific death signals, and translocated to the nucleus, where it induces DNA fragmentation leading to apoptosis [21]. AIF has a crucial role in the caspase-independent apoptotic pathway [21] and as such was chosen as our killing moiety for the chimeric proteins, termed GnRH-AIF.

We constructed, expressed, and highly purified GnRH-AIF chimeric proteins. We demonstrated the ability of the chimera to enter and specifically and very efficiently kill human solid cancer cells overexpressing GnRH-R. The GnRH-AIF chimeric proteins enter target cells by binding to the GnRH-receptor, which is overexpressed on the cell surface of solid tumors cells and undergoes internalization following this binding (receptor-mediated internalization). Most importantly, death induced by GnRH-AIF chimeric protein is caspase-independent. Finally, we demonstrated the high anti-tumor efficacity of the chimera, in a colon cancer organoid model. Our study shows the potential of a GnRH-AIF chimeric protein as a novel approach to treat solid cancers that overexpress GnRH-R, via a caspase-independent apoptotic pathway.

## 2. Materials and Methods

### 2.1. Construction of the Plasmids Encoding GnRH-AIF Chimeric Proteins

**Figure 1:**
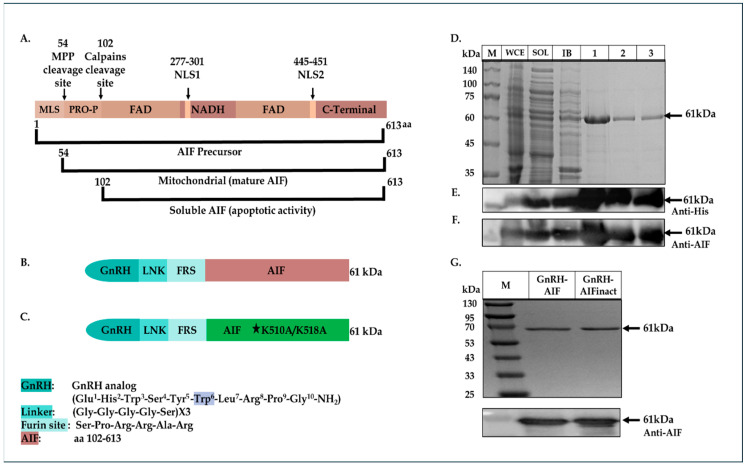
Expression and purification of GnRH-AIF chimeric proteins. (**A**) Schematic structure of human AIF protein indicating its different forms. (**B**,**C**) GnRH-AIF chimeric protein (**B**) and GnRH-AIFinact chimeric protein (**C**). Stars mark the point mutations introduced into AIF proteins. (**D**–**G**) Purification steps of GnRH-AIF chimeric protein: whole cell extract (WCE), soluble (SOL) and inclusion bodies (IB), pull-down fractions following affinity column purification (1); pull-down fractions after Q-Sepharose step (2); and after dialysis (3); and were separated on 12% SDS-PAGE and analyzed using Coomassie blue (**D**) and Western blot analysis using anti-AIF antibodies (**E**) or anti-His antibodies (**F**). Final preparation of GnRH-AIF and inactive GnRH-AIFinact chimeric proteins were separated on 12% SDS-PAGE and analyzed using anti-AIF antibodies (**G**). Arrows indicate size of the chimeric proteins. Protein markers on gels are marked by M. inact = inactive; MLS = mitochondrial leader sequence; NLS = nuclear localization signal; FAD binding domain = flavin adenine dinucleotide binding domain; NAD binding domain = nicotinamide adenine dinucleotide binding domain. FRS = Furin recognition site; PRO-P = pro-peptide; LNK = linker. The original Western blot figures can be found in Supplementary File S1.

A plasmid encoding the CD40-Smurf2 sequence [23,24] was cut with a BmtI and AgeI restriction enzyme removing the CD40 coding sequence and resulting in the vector fragment. The insert consists of the GnRH coding sequence, with tryptophan replacing glycine as the sixth amino acid, flanked by BmtI and AgeI (3′ end) restriction sites. GnRH-Smurf2 sequence was cut with EcoRI and Sac1 restriction enzymes, removing the Smurf2 coding sequence and resulting in the vector fragment. The human AIF protein sequence (amino acids 101-649) was generated and amplified by PCR using an AIFM1 clone as a template (clone number: BC139738, GE Healthcare Dharmacon Inc., Lafayette, CO, USA) with the following synthetic oligonucleotide primers:

5′-GAGGAATTCTCTCCACGGCGGGCACG TGGGCTGACACCAGAACAGAAAC-3′ (sense) and 5′-CACGAGCTCTCAGTCTTCATGAAT GTTGAATAGTTTGGC-3′ (antisense). The product fragment (containing also a Furin site before the AIF sequence, see Figure 1B below), cut with EcoRI and Sac1 restriction enzymes, was ligated into the vector fragment using Quick Ligation Kit (New England Biolabs, Ipswich, MA, USA), 3′ to the GnRH coding sequence, thus producing the plasmid encoding the GnRH-AIF chimeric protein.

As a negative control, a plasmid containing a known deactivating mutation (alanine substitutions of lysine at the positions 510/518) [25] in the DNA binding site of AIF was generated using PCR steps and the Gibson Assembly protocol (#E2611S, New England Biolabs). In addition, GnRH-AIFct, which encodes amino acids 353-613 of AIF, corresponding to its C-terminal domain and GnRH-AIFnt corresponding to the N-terminal region of AIF (amino acids 101–352), were generated using the same protocol. For the construction of the GnRH-AIF variants, two PCR reactions were conducted using the primers detailed in Table 1. The amplified products were verified using 1% agarose gel. The DpnI enzyme was added to the two products, and both products were used for the Gibson reaction. The coding sequences of the chimeric proteins GnRH-AIFinact, GnRH-AIFct, or GnRH-AIFnt were confirmed by sequencing analysis.

### 2.2. Construction of the Plasmids Encoding GnRH-Caspase-3 Chimeric Proteins

For comparison studies, we designed GnRH-Caspase-3 chimeric proteins with caspase 3, a pro-apoptotic protein of human origin, as an active moiety. This protein causes cell death via caspase-dependent apoptosis pathways.

The Smurf2 coding sequence was removed from the plasmid encoding the GnRH-Smurf2 sequence, as described above. The caspase 3 sequence was generated and amplified by PCR using the plasmid encoding the IL2-Caspase-3 [26] (lacking the pre-sequence) with the following synthetic oligonucleotide primers covering the whole coding sequence: 5′ CGGAATTCAACAGCTATAAAATGGATTA 3′ (sense) and 5′CCGCTCGAGTTAGTGGTA GAAGTACAGTTCTTTGG 3′ (antisense). The caspase 3 fragment, cut with EcoRI and Xho1 restriction enzymes, was ligated into the vector fragment containing the GnRH coding sequence using Quick Ligation Kit, thus producing the plasmid encoding the GnRH-Caspase-3 chimeric protein. The coding sequences of the chimeric proteins GnRH-Caspas-3 was confirmed by sequencing analysis.

### 2.3. Expression of GnRH-AIF and GnRH-Caspase-3 Chimeric Proteins

Following plasmid transformation into Rosetta strain *Escherichia coli*, the bacterial cultures were grown at 37 °C until an OD_600_ of 0.7–0.8. Expression was induced by adding 2 mM isopropyl-1-thio-D-galactopyranoside (IPTG, #16242352, New England Biolabs, Ipswich, MA, USA), and growth continued for additional 3 h. The cells were then centrifuged for 20 min at 4000× *g*, and the pellet was stored at −80 °C for 30 min. The frozen cells were thawed and suspended in lysis buffer (20 mM Tris-HCl, pH 8.0; 0.05 M NaCl; 0.2 mg/mL lysozyme; and 1 mM phenylsulfonyl fluoride) for 30 min at room temperature, followed by sonication. A sample was taken from the lysed cells and marked as whole cell extract (WCE). The rest of the cells were then centrifuged at 13,000× *g* for 30 min. The supernatant (marked as soluble fraction; SOL) was removed, and the pellet (containing inclusion bodies; IB) was suspended in denaturation buffer (6 M urea, 20 mM Tris-HCl, pH 8.8, 0.05 M NaCl, and 1 mM phenylsulfonyl fluoride) and continuously stirred for 90 min. The solution was cleared by centrifugation at 13,000× *g* for 60 min. The supernatant, containing the denatured proteins, was collected and marked as IB.

### 2.4. Purification of GnRH-AIF and GnRH-Caspase-3 Chimeric Proteins

The denatured chimeric proteins in 6 M urea were filtered, and a final concentration of 10 mM imidazole was added. The protein solution was loaded onto His-Trap columns (GE Healthcare, Sweden), which was pre-washed in the loading buffer (6 M urea, 20 mM Tris-HCl, pH 8.8, 0.05M NaCl, and 10 mM imidazole). To elute the desired protein, the column was washed with an increasing gradient of imidazole until a maximum concentration of 500 mM. Fractions containing the chimeric protein were pooled and slowly diluted 1:80 (vol/vol) in refolding buffer (20 mM Tris-HCl, pH 8.8, 2 mM β-mercaptoethanol (βME), 1 mM EDTA, 0.05 M NaCl, and 1 mM DTT) and then incubated at 4 °C for 72 h. The refolded chimeric protein solution was then subjected to ion exchange chromatography using a Q-Sepharose column (GE Healthcare, Upsala, Sweden) and using a SP-Sepharose column for GnRH-AIFnt (the chimeric protein is diluted in refolding buffer pH 6.5). Protein was eluted with linear gradient of 0.05–1 M NaCl in refolding buffer. Fractions containing the chimeric protein were pooled, and the protein preparation was dialyzed against phosphate buffered saline (PBS), aliquoted, and kept at −20 °C. All purification procedures were conducted using the FPLC system ÄKTA (GE Healthcare, Sweden).

### 2.5. Characterization of the GnRH-AIF Chimeric Proteins

#### 2.5.1. Protein Concentration

Protein concentration was measured according to the Bradford method, using a Bradford reagent (#5000006, Bio-Rad Laboratories, Inc., Berkeley, CA, USA) and a standard curve of BSA. Protein concentration was determined at a wavelength of 595 nm.

#### 2.5.2. Separation of Chimeric Proteins by Gel Electrophoresis

Samples from the various subcellular fractions (WCE, SOL, and IB) were separated on 12% SDS-PAGE gels and stained with Coomassie blue for visualization of the proteins.

#### 2.5.3. Western Blot Analyses

Protein samples were separated on SDS-PAGE gels and then electro-transferred onto a polyvinylidene fluoride (PVDF) membrane (Bio-Rad). The membrane was then blotted with one of the following antibodies: rabbit anti-AIFM1 (1:1000; ab32516, Abcam, Cambridge, UK); mouse anti-His (1:10,000; #27-4710-01, Amersham Pharmacia Biotech, Uppsala, Sweden); mouse anti-tubulin (1:10,000; ab7291, Abcam); mouse anti-lamin (1:1000, ab8983, Abcam); or rabbit anti-SF2 (1:1000, ab133689 Abcam). The secondary antibodies used were goat anti-rabbit or goat anti-mouse HRSP-conjugated (1:10,000, Jackson ImmunoResearch Laboratories Inc., West Grove, PA, USA). Band visualization was performed using the EZ-ECL chemiluminescence detection kit (Biological Industries, Beit HaEmek, Israel).

### 2.6. Cells Lines

All cell lines were obtained from the American Type Culture Collection (ATCC, Manassas, VA, USA). MCF-7 breast adenocarcinoma cells (ATCC-HTB-22), LNCaP prostate adenocarcinoma cells (ATCC-CRL-1740), Colo205 (ATCC-CCL-22), Colo320 (ATCC-CCL-220), SW48 (ATCC-CCL-231), and HCT115 (ATCC-CCL-225) colon adenocarcinoma cells were grown in RPMI 1640 medium (L0501, Biowest, Nuaille, France) supplemented with 10% fetal bovine serum (FBS; Merck, Darmstadt, Germany), 2 mM L-glutamine (Sartorius, Göttingen, Germany), and 100 units/mL penicillin (penicillin-streptomycin solution 100×; Biowest). A204 rhabdomyosarcoma cells (ATCC-HTB-82), T24P bladder urinary transitional cell carcinoma (ATCC-HTB-4), normal fibroblasts, (ATCC-PCS-420-013), lung A549 cells (ATCC-CCL-185), and HEK-293 renal adenocarcinoma cells (ATCC-CRL-1573) were grown in DMEM medium (01-055-1A, Sartorius) supplemented with 10% FBS, 2 mM L-glutamine, and 100 units/mL penicillin. All cells were maintained in flasks and grown in a highly humidified atmosphere of 5% CO_2_ at 37 °C. Expression levels of GnRH-R on the various cell lines are summarized in Table S1.

### 2.7. Cell Viability Assay

A total of 0.5 × 10^4^ cells/100 µL/well were seeded in 96-well plates, treated with the various GnRH-AIF chimeric proteins, GnRH-Caspase-3 chimeric proteins (positive control), or PBS, and incubated for 24/48/72 h. Cell viability was determined using the Cell Titer-Blue kit (G8080, Promega, Madison, WI, USA) according to manufacturer’s instructions.

In addition, Colo205 and SW48 cells treated as described above were incubated in the IncuCyte^®^ S3 Live-Cell Analysis System and tracked for up to 72 h. Images of each well were obtained every 2 h. The data were analyzed using IncuCyte^®^ S3 Software 2019A (Sartorius).

### 2.8. Confocal Microscopy

A total of 5 × 10^5^ Colo205 cells were seeded in 3 cm plates and treated with 1 µM GnRH-AIF or PBS and incubated for 24 h. Treated cells were washed with cold PBS and then fixated with 1 mL of 4% paraformaldehyde for 10 min at room temperature. Next, cells were treated with a permeabilization solution (0.1% TritonX100 in PBS) for 10 min. To block unwanted nonspecific interactions, the cells were treated with 1% BSA and 0.3 M glycine in PBST (PBS + 0.1% Tween) for 30 min. GnRH-AIF chimeric proteins were stained with rabbit anti-AIFM1 antibodies overnight (1:500) and visualized with secondary 555-anti Rabbit antibody (1:200, AB_3095431 Jackson ImmunoResearch Laboratories Inc., West Grove, PA, USA). The cells were incubated with 1:1000 DAPI (D9542, Sigma-Aldrich, St. Louis, MO, USA) for 10 min, and then the slides were covered with mounting buffer overnight and analyzed using confocal microscope (Nikon Eclipse NI DS-Qi2, 60X, Nikon Instruments Inc., New York, NY, USA).

### 2.9. Internalization of GnRH-AIF Chimeric Proteins Analyzed by Western Blot

A total of 2 × 10^6^ Colo205 cells were seeded in 6-well plates and treated with chimeric proteins or PBS for 24 h. Internalization of the chimeric proteins into target cells was examined by Western blot analysis of treated cell extracts using anti-AIFM1 antibodies (see above).

### 2.10. Subcellular Fractionation of Target Cells

A total of 3 × 10^6^ LNCaP cells were seeded in 10 cm plates and treated with the chimeric proteins or PBS and incubated for 24 h. Subcellular fractionation to cytosolic and nuclear fractions was performed using the RIME protocol [27]. Protein concentration was measured according to the Bradford method to ensure equal loading of nuclear and cytoplasmatic fractions.

### 2.11. Cell Cycle Analysis by Propidium Iodide Staining

A total of 2 × 10^6^ Colo205 cells were seeded in 3 cm plates and treated with 1 µM GnRH-AIF or PBS and incubated for 24 h. The cells were collected, counted, and washed twice with PBS and then treated with Nicoletti buffer (50 µg/mL propidium iodide, 0.1% sodium citrate, 0.1% TritonX100), for 1 h at 4 °C in the dark. Cell cycle analysis of the cells was performed using flow cytometer analyzers (C6 Plus Flow Cytometer, Biosciences, Denver, CO, USA) and analyzed using the FCS Express7 program.

### 2.12. Annexin V Assay

A total of 2 × 10^6^ Colo205 cells were seeded in 6-well plates treated with 1 µM GnRH-AIF or PBS and incubated overnight. Cells were harvested, washed with PBS, and resuspended in an Annexin-binding buffer at a concentration of 1 × 10^6^ cells/mL. The samples were stained with 5 µL of Annexin V and 5 µL of 7AAD and then incubated for 15 min at RT (#62700-80, Annexin V APC Kit, Biogems, Westlake Village, CA, USA). Following incubation, cells were diluted with 400 mL of Annexin-binding buffer and analyzed by flow cytometry immediately. Unstained and single-stained cells were used for compensation and statistics. Cells stained with Annexin represent early apoptotic cells. Double-stained cells represent late apoptotic cells, and cells stained with 7AAD represent necrotic cells. Samples were analyzed with a LSR II flow cytometer (Biosciences, Denver, CO, USA).

### 2.13. Real-Time PCR Analysis of Apoptosis-Related mRNAs

A total of 1.0 × 10^6^ Colo205 cells were seeded in 3 cm plates treated with GnRH-AIF (1 µM) or PBS and incubated for 48 h. Total RNA was extracted from cells using a commercial kit (#GZXD200, GENEzol™ TriRNA Pure Kit, Geneaid, New Taipei City, Taiwan). RNA concentrations were determined using a NanoDrop spectrophotometer (Thermo Scientific, Waltham, MA, USA). One µg of each RNA sample was reverse transcribed using a reverse transcription kit (qScript cDNA Synthesis Kit, Quantabio, Beverly, MA, USA, Cat# 95047-100-2). The resulting cDNA was diluted in DNase-free water (1:10) before quantification by real-time quantitative PCR. Individual mRNA levels were quantified using real-time PCR. Each sample contained 2 µL of cDNA, 1 µL of primers, 5 µL of SYBR^®^ Green, and 3 µL of H_2_O in a total volume of 10 µL per sample. The primers used are listed in Table 2. The data were analyzed using the primer express program (Applied Biosystems, Foster City, CA, USA). All data are expressed as the ratio between the expression level of the target gene mRNA and that of actin.

### 2.14. In Vitro Caspase 3 Activity Assay

A total of 0.5 × 10^4^ cells/100 µL/well of Colo205 were seeded in 96-well plates and cultured in medium with or without 20 µM zVAD-FMK (sc-3067, Santa Cruz Biotechnology Inc., Dallas, TX, USA) for 5 h and then were treated with GnRH-Caspase-3 (7 µM) and GnRH-AIF (1 µM) and incubated for 48 h. Caspase 3 activity within the cells was assessed by a Apo-ONE^®^ Homogeneous Caspase 3/7 Assay Kit (Promega), and caspase 3 activity was analyzed using a FLU Ostar plate reader at the following wavelengths: excitation/emission—485/520 nm. Results are shown as fold increases in caspase 3 activity in the treated cells compared to control cells and were normalized to number of cells.

### 2.15. Gel Retardation Assay

DNA-binding activity of GnRH-AIF chimeric proteins was determined by gel retardation assays. A total of 2.5 μg of 1 kb DNA ladder (DM015-R500, Hylabs, Rehovot, Israel) was incubated for 30 min at room temperature with GnRH-AIF proteins and then analyzed by agarose gel electrophoresis (1%) in the presence of ethidium bromide to visualize the DNA.

### 2.16. Nuclease Activity Assays

Assays were performed by mixing 250 ng of a double-stranded, supercoiled pET-28 plasmid or 500 ng of genomic DNA as substrate with 12 μg of GnRH-AIF chimeric proteins, GnRH-AIFinact, and GnRH-Caspase-3 (or 1 IU of DNase for 1 min and 5 min) in 20 mM Tris, having a pH 8.0 and a final volume of 10 μL, with 0.1 mM CaCl_2_ and 1 mM MgCl_2_. The samples were incubated for 20 min at 37 °C, after which they were mixed with 6× DNA loading dye (Thermo Scientific). Samples were then loaded onto 0.8% agarose gel with ethidium bromide and run for 1 h at 100 V.

### 2.17. DNA Laddering

A total of 1.5 × 10^6^ Colo205 cells were seeded in 1 mL of medium and treated with 1 µM GnRH-AIF or PBS for 24 h. The cells were then collected and washed twice with PBS, and DNA was extracted using the GenElute Blood Genomic DNA Kit (NA2010-1KT, Sigma-Aldrich). A total of 10 µg of DNA of each sample was loaded on a 1.5% agarose gel.

### 2.18. Immunoprecipitation and Co-IP Assay

AIF was immunoprecipitated using nuclear fractions of treated and control Colo205 cell extract, 1 µg of rabbit monoclonal anti-AIF antibody (ab32516, Abcam, Cambridge, UK), and 30 µL of Protein A/G beads (sc-2003, Santa Cruz Biotechnology, Dallas, TX, USA) The antibodies were incubated under agitation with Protein A/G beads for 4 h, and then the nuclear fraction was added to each sample and incubated overnight under agitation at 4 °C. The beads were washed with PBS to remove non-specific binding. Proteins were eluted by the addition of 40 µL SDS loading buffer (X2) and incubated for 10 min at 95 °C; 20 µL of each sample was separated on a SDS-PAGE gel, transferred to a nitrocellulose membrane, and probed with anti-ENDOG (#22148-1-AP, Proteintech Group, Inc., Munich, Germany), rabbit anti-AIFM1 (1:1000; ab32516, Abcam), or anti-lamin (1:1000, ab8983, Abcam).

### 2.19. Knockdown of Endonuclease G and Cyclophilin A (PPIA) by siRNA

esiRNA (# EHU-904001, Sigma-Aldrich) targeting human ENDOG gene transcripts was used for the knockdown of ENDOG. esiRNA (#EHU107101, Sigma-Aldrich) targeting human PPIA gene transcripts was used to knockdown PPIA. LNCaP cells were transfected with 240 µL of reduced serum medium (# 31-985-070, Opti-MEM™, Thermo Fisher Scientific), 3 µL of TransIT-X2 (#MC-MIR-6000, Mirus, Marietta, GA, USA), and 3 µL of a 14 µM siRNA stock solution (20 nM final concentration per well), which were mixed and incubated at room temperature for 30 min. In a 6-well plate, 245 µL of the mix were added in each well, and then 2 × 10^5^ cells/1.5 mL were seeded in RPMI medium supplemented with 10% serum (without antibiotics). A total of 24 h later, the media was replaced with a complete growth medium, and the cells were seeded in 96-well plates for 4 h prior to treatment. Wild type and cells after knocking down were treated with various concentrations of GnRH-AIF or GnRH-Caspase-3 and incubated for a further 24 h. Cell viability was determined using the Cell Titer-Blue kit (G8080, Promega) according to manufacturer’s instructions. Validation of the knockdown was confirmed using primers of these genes (see Table 2) and real-time PCR (see above), as well as at the protein level (Figure S2) by Western blot analysis using anti-ENDOG antibodies (see above).

### 2.20. Organoid Culture

Colorectal cancer (CRC) as well as healthy, adjacent colon tissues were obtained from a colorectal carcinoma patient (male, aged 85) and a gastric carcinoma patient (female, aged 15) following surgery under the Helsinki authorization #HMO-0921-20. Biopsies were minced into 2–3 mm fragments and processed within 90 min in digestion medium (CRC—Advanced DMEM/F-12 supplemented (#12634010, Gibco™ Thermo Fisher Scientific Inc., USA) with 1% Anti-Anti, 1% HEPES (1 M solution), 1% GlutaMAX, 1 mg/mL collagenase type II, 10 µg/mL hyaluronidase, and 10.5 µM Y-27632 for 60 min at 37 °C with gentle agitation; Healthy colon—10 mL 0.01 M EDTA in PBS for 90 min at 4 °C and are then mechanically tritured to extract intestinal crypts). Digestion is stopped by the addition of Advanced DMEM/F-12 supplemented with 0.1% BSA. CRC samples are then filtered through a 100 µm mesh. Samples are centrifuged at 250× *g* (CRC) or 200× *g* (Healthy colon) for 5 min at 4 °C. Red blood cells are lysed if necessary.

The resulting pellets are resuspended in 50-100% ice-cold growth factor-reduced Matrigel (CRC—1.6 × 10^6^ cancer cells/mL; Healthy colon—8 crypts/10 uL) and distributed in pre-heated 24-well plates (30 μL domes). Plates were inverted and incubated at 37 °C for 20 min. Then, 500 µL of growth medium was added per well. CRC growth medium included the following: Advanced DMEM/F-12 (#12634010, Gibco™ Thermo Fisher Scientific Inc., USA, 1% Anti-Anti, 1% HEPES, 1% GlutaMAX, 500 nM A83-01(SML0788 Merck), 0.02X B27 without vitamin A (17504044, Gibco™), 1.25 mM N-acetyl-L-cysteine (A9165, Sigma-Aldrich), 10 mM nicotinamide (N0630, Sigma-Aldrich), 1 µM PEG2 (2296/10 R&D Systems)), 100 µg/mL Primocin (ant-pm-2 Invivogen), 50 ng/mL recombinant human EGF (Peprotech), 100 ng/mL recombinant human Noggin (Peprotech), 500 ng/mL recombinant human R-spondin-1 (Peprotech), 0.3 µM SB202190 (S7067 Sigma-Aldrich), and 10.5 µM Y-27632 (10005583 Cayman Chemicals). Healthy colon medium included the following: IntestiCult™ Organoid Growth Medium (#06010, Stem Cell Technologies, Vancouver, BC, Canada) supplemented with primocin and 10.5 µM Y-27632.

Organoids formed typically within four days, and the medium was refreshed twice a week. Organoids were passaged by mechanical disruption of the Matrigel, washing in Advanced DMEM/F12 medium and resuspension in ice-cold GFR Matrigel.

For cell viability assay, organoids were seeded on an 8-well IBIDI slide at a concentration of 5 organoids per well in 12 µL Matrigel and cultures in complete medium supplemented with various concentrations of GnRH-AIF (1, 1.5, and 3 µM) or PBS for three days. Cultures were then incubated with propidium iodide (PI) and Hoechst 33,342 according to the manufacturer’s instructions. Images were acquired on a spinning disk confocal microscope (Yokogawa W1 Spinning Disk) (4× and 20× objective), and analysis was performed using the NIS program (NIS Elements software #46002). For all experiments, three independent experiments were conducted. Areas of individual organoids were measured and exported as Excel files. Hoechst 33,342 and propidium iodide (PI) channels were separated and loaded as matched channels. Then, the total cell nuclei area (Hoechst 33342) and PI staining area were calculated for each organoid and exported to a spreadsheet. The cell death was calculated by dividing PI area per nuclei area. Results are shown as the percentage of organoid deaths calculated for treated organoids as compared to control (treated with PBS only).

### 2.21. Statistical Analyses

Results of cell viability assays are the average of 3–6 independent biological experiments ± SD. The results of the Western blot analyses were evaluated by densitometry using the Image Lab Software version 5.2.1 (Bio-Rad). Statistical analysis was performed using Microsoft Excel 2017 and an unpaired, two-tailed t test was used to determine *p* values.

## 3. Results

### 3.1. Construction and Expression GnRH-AIF Chimeric Proteins

The aim of this study was to construct a chimeric protein able to target cancer cells overexpressing GnRH-R and induce caspase-independent death. To design the chimeric protein, we used a GnRH analog, a small peptide of 10 amino acids, as the specific targeting moiety (GnRH–trp6; tryptophan was substituted for glycine in the sixth position) [28]. The killing moiety of the chimera is composed of the human AIF protein lacking the first 101 residues at the N-terminal (the MLS and the pro-peptide) [29]. The removal of these sequences prevents the protein from entering and anchoring into the IMM of the mitochondria (Figure 1A). In addition, a consensus recognition sequence for Furin protease [30] was inserted in an attempt to induce the cleavage and separation of the AIF moiety from the targeting motif once the chimera enters the target cell. Furthermore, a poly-linker was introduced between GnRH and the killing component of the chimeric proteins to enable these molecules to fold properly. The poly-linker is composed of (gly_4_ser_2_)X3, similar to the polylinker used in single chain antibodies [31,32]. The coding sequence of the plasmid was confirmed by sequence analysis. Figure 1B demonstrates the schematic structure of the final chimeric protein termed GnRH-AIF. The chimeric protein was expressed in several *E. coli* expression systems, and various subcellular fractions were prepared: whole cell extract (WCE), soluble fraction (Sol), and inclusion bodies (IB), separated on SDS-PAGE, and characterized both by Coomassie blue staining, and by Western blot analyses, using anti-AIFM1 and anti-His antibodies. Figure 1D–F confirmed the expression of GnRH-AIF chimeric protein (~61kDa), as well as the identity of the chimera. In addition, we constructed a GnRH-AIF chimeric protein with mutations changing lysine to alanine at amino acid positions 510 and 518 (mut L510A and mut L518A) in the human AIF component, resulting in a chimeric protein termed GnRH-AIFinact. These mutations were reported to abolish the ability of AIF to bind to the DNA and to induce apoptosis [25]. Figure 1C demonstrates the schematic structure of the GnRH-AIFinact chimeric protein. The inactive protein was expressed and purified using the same protocol as the authentic chimeric protein, to minimize differences between the chimeric proteins (Figure 1G). After calibrating the different conditions, the most successful expression system was as follows: expression in the Rosetta bacterial strain until OD = 0.7–0.9, induction with 2 mM IPTG, and growth at 37 °C for 3 h.

### 3.2. Purification of GnRH-AIF Chimeric Proteins

The chimeric proteins were cloned with His-tag (Hisx6) and so could be purified using a nickel (Ni+) affinity column. The expressed GnRH-AIF chimeric proteins accumulated in the IB subcellular fraction of the bacterial host (Figure 1D). Therefore, the IB were dissolved in a 6 M urea-based buffer and loaded onto the affinity column. Elution was performed using an increasing concentration gradient of imidazole. Peak fractions were collected and analyzed using Coomassie blue and Western blot analyses and were taken for refolding for 72 h in a buffer and ratio calibrated that was found to be 1:80 *v*/*v*. Following refolding, the chimeric protein was loaded onto a Q-Sepharose column, and elution was performed via an ascending gradient of NaCl concentration. Peak fractions were collected; the buffer was exchanged to PBS by dialysis and defined as the GnRH-AIF chimeric proteins for all future experiments. Figure 1D–F demonstrates the purification steps of GnRH-AIF chimeric proteins by SDS-PAGE (Figure 1D) including Western blot analyses using both anti-His and anti-AIF antibodies for the GnRH-AIF chimeric protein (Figure 1E,F). Figure 1G demonstrates the final, highly purified GnRH-AIF and GnRH-AIFinact chimeric protein preparations, and its identity was confirmed by Western blot analysis using anti-AIFM antibodies.

### 3.3. Internalization of GnRH-AIF into Target Cancer Cells

**Figure 2:**
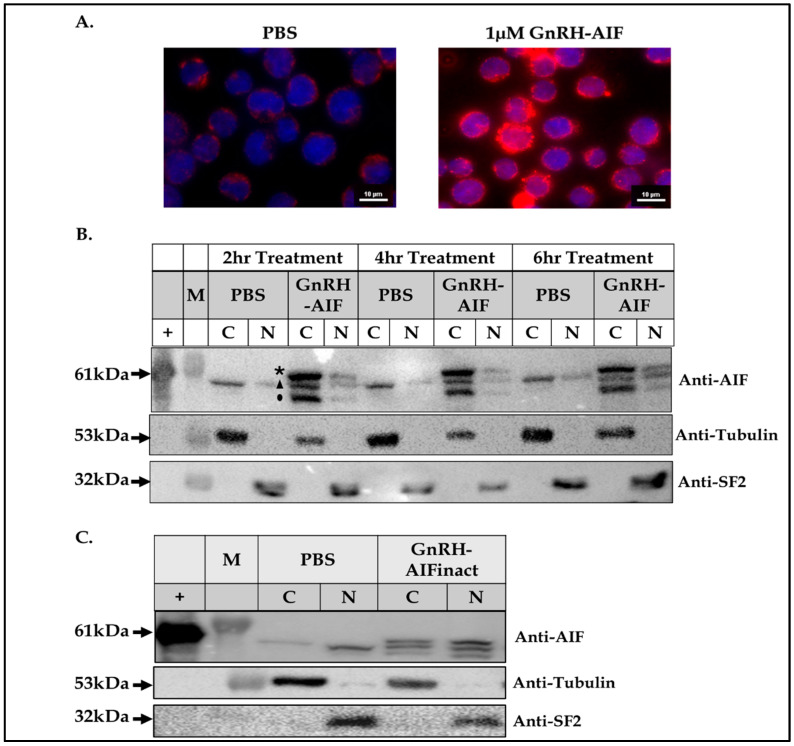
Internalization of GnRH-AIF chimeric protein into human target cancer cells. Confocal microscopy images of Colo205 cells treated with GnRH-AIF (1 µM, **right** image) compared to control (PBS, **left** image). GnRH-AIF chimeric proteins were detected using anti-AIF antibodies and 555-anti rabbit (red). DAPI was used to stain the nucleus (blue) (magnification 60×) (**A**). Following sub-fractionation of LNCaP cells treated with GnRH-AIF chimeric protein for 2, 4, and 6 h (**B**), or GnRH-AIFinact chimeric protein for 24 h (**C**), the nuclear and cytosolic sub-fractions were analyzed using anti-AIF, anti-Tubulin, and anti-SF2 antibodies. C = cytosol; N = nucleus; inact = inactive; + = positive control. * Exogenous GnRH-AIF chimeric protein (Full length) ▲ Endogenous AIF ● Exogenous AIF after Furin cleavage. The original Western blot figures can be found in Supplementary File S1.

The internalization kinetics of the GnRH-AIF chimeric protein into colon adenocarcinoma cells (Colo205 cells), known to overexpress the GnRH-R, was demonstrated using confocal microscopy and visualized with secondary fluorescent antibodies. Colo205 cells were incubated with GnRH-AIF for 24 h, and we followed the intensity of the fluorescent staining within the treated cells (Figure 2A). Since human cells express endogenous AIF, low levels of AIF were detected within untreated cells (PBS, Figure 2A, left image). However, treated cells were significantly more positive for fluorescent staining, which implies that the chimeric protein entered the target cancer cells (Figure 2A, right image). We also examined the internalization kinetics of GnRH-AIF into target cancer cells using Western blot analysis of extracts of treated cells. LNCaP cells (prostate cells) were incubated with the chimeric protein for 2, 4, and 6 h and subjected to sub-fractionation. The GnRH-AIF chimeric protein was detected both in the cytoplasm and in the nucleus as early as two hours after the start of treatment, at the expected molecular weight (Figure 2B). In addition, we observed two additional bands corresponding to the endogenous AIF protein and to the chimeric protein after cleavage at the Furin site. Following expression and purification of the inactive mutant GnRH-AIFinact chimeric protein, we incubated it with LNCaP cells. As seen in Figure 2C, the inactive chimeric protein entered target cancer cells and appeared as the same band size as the active chimeric protein. These experiments confirmed the ability of GnRH-AIF chimeric proteins to induce internalization into target cells upon binding, thus establishing again the feasibility of using the GnRH-GnRH-R as an efficient targeting system, similar to our previous studies using other GnRH-based chimeric proteins.

### 3.4. Efficacy of Death Induced by GnRH-AIF Chimeric Proteins

**Figure 3:**
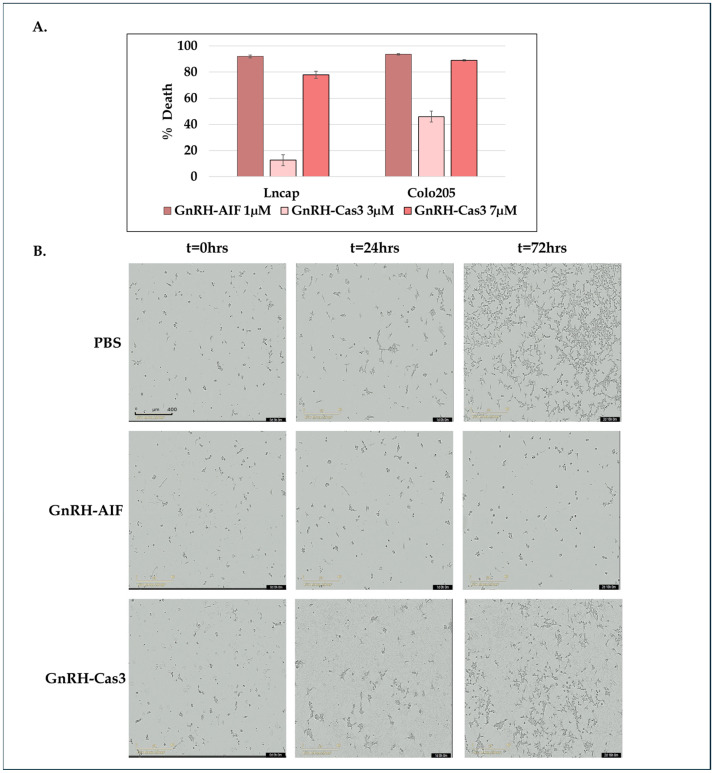
Efficacy of death induced by GnRH-AIF chimeric proteins. Colo205 and LNCaP cells are treated with GnRH-AIF (1 µM), GnRH-Caspase-3 (1 µM or 7 µM), or PBS and incubated for 72 h. Results are shown as percentage of cell death calculated for treated cells as compared to control cells (treated with PBS only). Results are the average of 3–6 repeats ± SD (**A**). SW48-positive control cells were treated with 1 µM GnRH-AIF and 7 µM GnRH-Caspase-3 or PBS and documented by the Incucyte machine for 72 h. Microscope images were taken at the beginning (t = 0) and then at 24 and 72 h. (magnification 10×) (**B**). Cas3 = caspase 3; t = time point.

In order to test whether the chimeric proteins were biologically active after expression and purification, we tested for their effect on cell viability of human colon and prostate cancer cells [28]. As a positive control for the experiment, we used the chimeric protein GnRH-Caspase-3 [33] and our unpublished data. All cell lines were treated with GnRH-AIF (1 µM), GnRH-Caspase-3 (3 µM and 7 µM), and PBS (control) for 72 h. Treatment of Colo205 and LNCaP target cells with the GnRH-AIF chimeric protein caused high levels of cell death (about 90%) after 72 h treatment (Figure 3A).

The positive control protein, GnRH-Caspase-3, caused 80–90% cell death at the same time point. However, while GnRH-AIF caused high levels of cell death at lower concentrations (1 µM), the concentration of GnRH-Caspase-3 causing similar death levels was about 7-fold higher (when calculated on a MW basis), so in fact it may be less potent. Thus, the GnRH-AIF chimeric protein is a highly effective molecule for causing cell death of human cancer cells overexpressing the GnRH-R.

Similar effects were observed when testing the biological activity of GnRH-AIF and GnRH-Caspase-3 chimeric proteins via live imaging (Figure 3B). SW48 colon cancer cells were treated with GnRH-AIF (1 µM) and GnRH-Caspase-3 (7 µM) or PBS as negative control, incubated for 72 h, and imaged every 2 h using the Incucyte system. While the initial images showed similar levels of viable cells, after just 24 h, proliferation was suppressed in cells treated with both chimeric proteins compared to the control (PBS). However, it appears that the cell death induced by GnRH-AIF was more rapid when compared to cells treated with GnRH-Caspase-3, similar to the results obtained by the viability assays.

### 3.5. GnRH-AIF Chimeric Protein Kills Target Cancer Cells in a Dose- and Time-Dependent Manner

**Figure 4:**
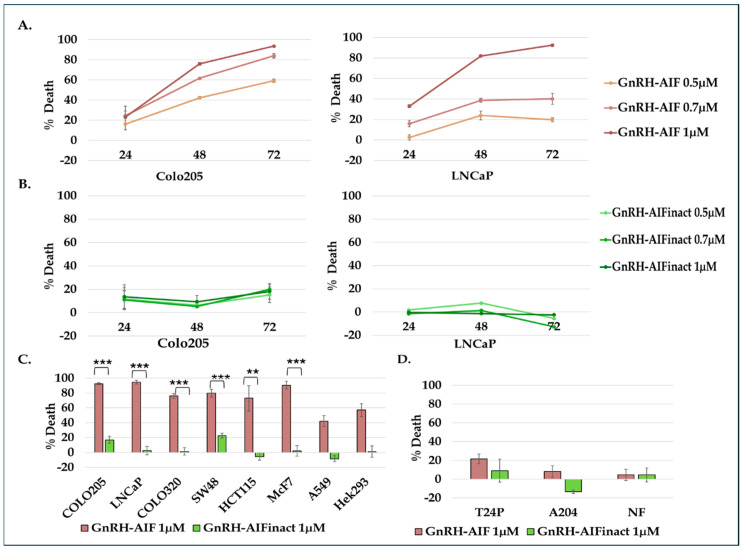
GnRH-AIF chimeric protein kills target cancer cells in a dose- and time-dependent manner. Colo205 and LNCaP cells were treated with various doses of GnRH-AIF and GnRH-AIFinact (0.5, 0.7, and 1 µM) and incubated for 24, 48, or 72 h demonstrating a dose- and time-dependent cell death (**A**,**B**). Simultaneously, Colo205, LNCaP, MCF-7, SW48, Colo320, HEK293, and A549 were treated with GnRH-AIF (1 µM) and GnRH-AIFinact mutant chimera (1 µM) for 72 h and tested under the same conditions (**C**). A204 cells, normal fibroblasts, and T24P cancer cells lacking GnRH-R expression were treated with GnRH-AIF or PBS (**D**). Results (**A**–**D**) are presented as percentage of cell death calculated for treated cells as compared to control cells (treated with PBS only). All results are the average of 3-6 repeats of the experiments ± SD. *p* value ≤ 0.01 = **; *p* value ≤ 0.001 = ***; inact = inactive.

Next, we checked whether activity of the GnRH-AIF was dose- and time-dependent. Colo205 and LNCaP cells were treated with increasing doses of the GnRH-AIF and the GnRH-AIFinact chimeric proteins (0.5, 0.7, and 1 µM) or PBS (control) for 24, 48, and 72 h. As demonstrated in Figure 4A, GnRH-AIF chimeric proteins induced a linear dose- and time-dependent increase in the percentage of cell death.

GnRH-AIF causes very rapid cell death. As early as 48 h post-start of treatment, percentage cell death was about 77% at a very low concentration (1 µM). As seen in Figure 4B, treatment of LNCaP and Colo205 cells with the GnRH-AIFinact chimeric protein did not kill the cells, demonstrating that the killing function of the active chimeric protein is due to the AIF-killing component of the chimeric protein.

### 3.6. Effect of GnRH-AIF Chimeric Proteins on Different Human Cancer Cells Lines Overexpressing the GnRH-R and Specificity of Its Activity

We tested additional human target cancer cells overexpressing the GnRH-R, related or unrelated to the reproductive system. Figure 4C shows the GnRH-AIF chimeric proteins’ (1 µM) ability to induce high levels of cell death in a variety of human cancer cells, including additional human colon cancer cells (Colo320 and HCT-115 cells), breast cancer cells (MCF7 cells), lung cancer cells (A549 cells), and cervical cancer cells (Hela cells). However, the amount of cell death induced by the GnRH-AIF chimeric protein varied considerably between cell lines, ranging from ~40 to 95% cell death at 72 h post-start of treatment. This is most likely due to differences in the level/amount of GnRH-R expressed on the various human cancer cell lines (Table S1 and [28,33]) and/or on the level of endogenous AIF expressed in these cell lines. In addition, other cellular factors may influence the efficacy of GnRH-AIF-induced apoptosis such as additional unknown mutations in proteins/components in different human cancer cells involved in caspase-independent apoptosis pathway(s).

Human fibroblast, A204 rhabdomyosarcoma cancer cells, and T24P bladder urinary transitional cell carcinoma cells, served as negative control cell lines, as these cells do not express the GnRH-R and had tested negative in previous studies [18,28,33]. In every experiment performed on target cell lines, we conducted tests under the same conditions, side by side, for the non-target cells, to test for specificity. GnRH-AIF chimeric proteins did not kill non-target human cancer cells (Figure 4D). In summary, the GnRH-AIF chimeric protein caused the cell death of both hormone-dependent and non-hormone-dependent cancer cells lines effectively, without affecting non-target cells lacking the expression of the GnRH-R.

### 3.7. Cell Death Induced by GnRH-AIF Is Dependent on the C-Terminal Domain of AIF

**Figure 5:**
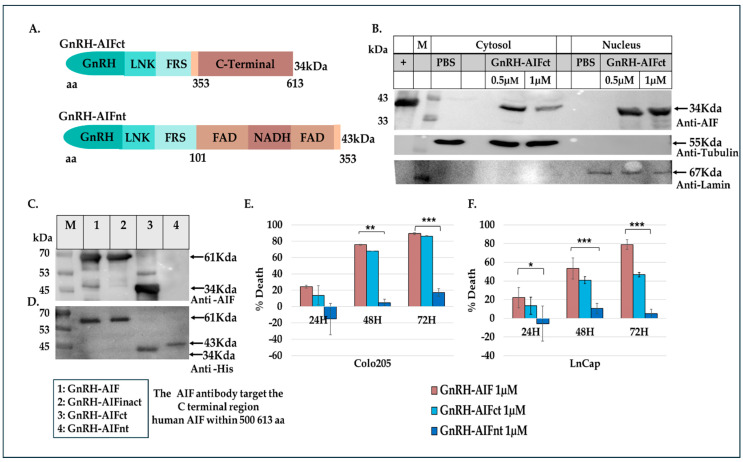
Cell death induced by GnRH-AIF chimeric protein is dependent on the c-terminal domain of AIF. Schematic structure of GnRH-AIFct and GnRH-AIFnt chimeric proteins (**A**). Following sub-fractionation of cells treated with the GnRH-AIFct chimeric protein for 24 h, the nuclear and cytosolic sub-fractions were analyzed using anti-AIFM1 and anti-Lamin or anti-Tubulin antibodies, respectively; (**B**). Following production, GnRH-AIF, GnRH-AIFinact, GnRH-AIFct, or GnRH-AIFnt chimeric proteins were separated on 12% SDS-PAGE and analyzed using anti-AIF (**C**) or anti-His antibodies (**D**). Colo205 and LNCaP cells were treated with GnRH-AIF, GnRH-AIFct, or GnRH-AIFnt and incubated for 24, 48, or 72 h. Results are presented as percentage of cell death calculated for treated cells as compared to control cells (treated with PBS only). All results are the average of 3 experiments ± SD. (**E**,**F**). *p* value ≤ 0.05 = *; *p* value ≤ 0.01 = **; *p* value ≤ 0.001 = ***. C = cytosol; N = nucleus; FRS = Furin recognition site; LNK = linker. The original Western blot figures can be found in Supplementary File S1.

The AIF protein comprises three domains: the N-terminal domain, the flavin adenine dinucleotide (FAD)-binding domain, and the C-terminal domain (Figure 1A). A previous study identifies a novel AIF transcript, called AIFshort (AIFsh) corresponding to the C-terminal part of AIF [34]. AIFsh provokes caspase-independent cell death, indicating that the pro-apoptotic activity of AIF resides in its C-terminal domain. Therefore, we constructed and designed a chimeric protein variant of GnRH-AIF, termed GnRH-AIFct, which included amino acids 353-613 of AIF, corresponding to its C-terminal domain, to confirm that the apoptotic function of the GnRH-AIF chimeric protein is indeed dependent on its C-terminal domain (Figure 5A).

First, we tested the ability of the GnRH-AIFct variant to enter GnRH-R expressing cancer cells and to reach the nucleus. Colo205 cells were incubated with GnRH-AIFct for 24 h. As seen in Figure 5B, this variant chimeric protein successfully entered target cells. Following sub-fractionation, GnRH-AIFct chimeric protein was found to be located within the nucleus, as was the original GnRH-AIF chimeric protein (Figure 5B). We also produced an additional variant corresponding to the N-terminal region of AIF (amino acids 101–352)-termed GnRH-AIFnt chimeric protein. Both variant chimeric proteins were produced and purified using the protocol established for GnRH-AIF chimeric proteins. This approach aimed at reducing differences among the tested chimeric proteins, as illustrated in Figure 5C,D. The GnRH-AIFnt chimeric proteins did not appear on the Western blot using the AIF antibodies because these antibodies bind to the C-terminal site; however, it was detected with the anti-His antibodies, confirming that we successfully purified GnRH-AIFct and the GnRH-AIFnt chimeric proteins.

Colo205 and LNCaP cells were treated with 1 µM of either GnRH-AIF, GnRH-AIFct, GnRH-AIFnt, or PBS (control) for 24, 48, and 72 h. As seen in Figure 5E,F, the GnRH-AIFct chimeric protein variant mimicked the biological activity of the original GnRH-AIF chimeric proteins. In contrast, GnRH-AIFnt chimeric protein did not kill the cells. Our finding confirmed that the killing function of the active GnRH-AIF chimeric protein is due to the C-terminal domain of AIF protein.

### 3.8. GnRH-AIF Chimeric Proteins Cause Cell Death of Target Cells via Apoptosis

GnRH-AIF was designed and produced to target and eliminate cancer cells that overexpress GnRH-R. However, we intended to kill these cells via apoptosis to avoid both tissue damage and a non-specific systemic response. Therefore, we examined both cell death and apoptosis in target cancer cells using several assays to ensure that the killing activity of GnRH-AIF chimeric protein was achieved via apoptosis.

**Figure 6:**
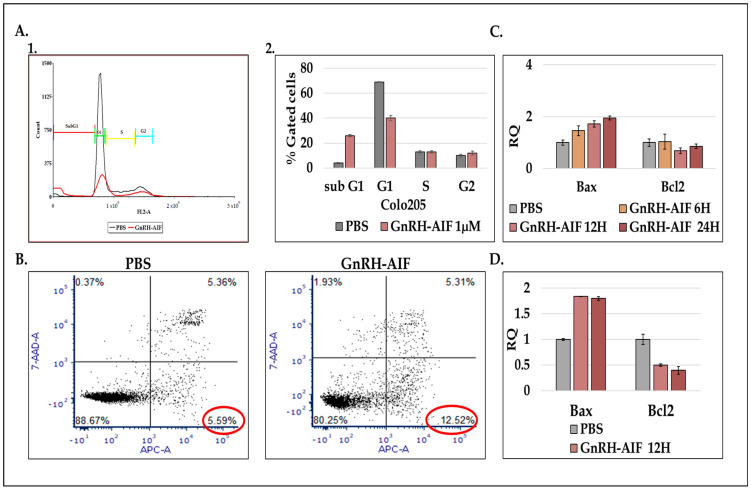
GnRH-AIF chimeric proteins cause cell death via apoptosis. Colo205 cells were treated with GnRH-AIF (1 µM) or PBS for 24 h and stained with PI and FACS-analyzed as described in methods (**left image A1**); Quantification of cells in cell cycle phases, marking the subG1 phase (**right image A2**). Black and red line indicate cell cycle of untreated and treated cells with GnRH-AIF, respectively (**A**). Colo205 cells were treated with GnRH-AIF (1 µM) for 24 h, stained with Annexin V and 7AAD and FACS-analyzed. Circles indicate the double-stained cells representing early apoptotic cells (**B**). Relative quantification (RQ) results using real-time PCR analysis for cellular mRNA human Bax and human Bcl2 expression levels upon treatment with GnRH-AIF (1 µM) chimeric proteins for 6, 12, or 24 h, as compared to untreated cells (PBS) in SW48 colon cancer cells (**C**) and LnCap prostate cancer cells (**D**). All results are the average of 3 experiments ± SD.

Colo205 cells were treated with 1 µM GnRH-AIF for 24 h and stained with PI to test its effect on the cell cycle of the treated cells. As seen in Figure 6A, following treatment, there was a 9-fold increase in the sub-G1 population compared to control cells.

Further evidence that GnRH-AIF chimeric proteins caused apoptotic death was obtained using the Annexin-V assay. Colo205s were incubated with GnRH-AIF chimeric proteins or PBS as a negative control for 24 h and then stained with Annexin and 7-AAD. Viable cells were then analyzed by flow cytometry. As can be seen in Figure 6B, 12% of the cells were identified as early apoptotic cells after 24 h incubation with the GnRH-AIF chimeric proteins, compared to 5% for negative control, which indicated a 2.5-fold increase in the apoptotic cell population.

Next, we tested the effect of the chimeric proteins on the expression of apoptosis-related protein, Bax, a central player of apoptosis, and Bcl-2, a major anti-apoptotic protein, at the mRNA level. SW48 and LNCaP human cancer target cells were treated with GnRH-AIF chimeric proteins for 6, 12, and 24 h, and then total mRNA was extracted and analyzed by real-time PCR. As can be seen in Figure 6C,D, when treating colon and prostate cancer cells, cellular mRNA levels of Bcl2 decreased in response to treatment while cellular mRNA levels of Bax increased in a time-dependent manner in both cell lines. The increase in the pro-apoptotic protein (Bax) in comparison to the untreated sample was 1.8-fold. The decline in the anti-apoptotic protein (Bcl2) was 2.5-fold. These results show that GnRH-AIF chimeric proteins affect the expression of both major pro- and anti-apoptotic proteins, disturbing their balance/ratio within the cells and finally leading toward apoptosis.

### 3.9. GnRH-AIF Induces Caspase-Independent Apoptotic Death

**Figure 7:**
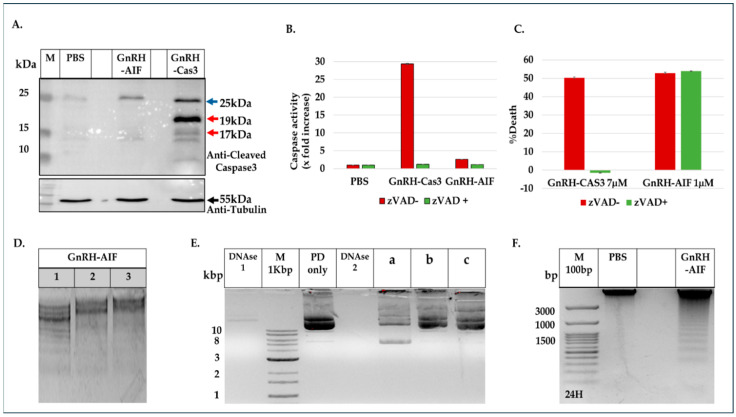
GnRH-AIF chimeric proteins induce caspase-independent apoptotic death. Colo205 cells were incubated with GnRH-AIF (1 µM) or GnRH-Caspase-3 (7 µM) for 24 h. Cell lysates were extracted and analyzed by Western blot analyses using anti-cleaved caspase 3 and anti-tubulin as a loading control. Red arrows indicate the large and small subunits of caspase 3 (after cleavage), and the blue arrow indicates the endogenous caspase 3 protein (before cleavage) (**A**). Colo205 cells were cultured in medium with or without 20 µM z-VAD-FMK for 4 h and then treated with GnRH-AIF (1 µM) or GnRH-Caspase-3 (7 µM) for 48 h (**B**). Experiments were conducted in parallel with cell viability assays (**C**). Results are shown as fold increases in caspase 3 activity in the treated cells compared to control cells and were normalized to number of cells. Results are the average of 3 experiments ± SD (**B**,**C**). DNA-binding activity of GnRH-AIF chimeric proteins was determined by a gel retardation assay. A total of 3 μg of 1 kb DNA ladder sample were incubated with GnRH-AIF chimeric protein for 30 min and analyzed by agarose gel electrophoresis (1%) (**D**). Double-stranded supercoiled pET-28 (+) plasmid substrate (250 ng) was mixed and incubated with GnRH-AIF (12 μg) (a), GnRH-AIFinact (b), GnRH-Caspase-3 (c), or 1 IU of DNAse for 1 min (DNAse 1) or 5 min (DNAse 2), incubated for 20 min at 37 °C, and analyzed by agarose gel electrophoresis (0.8%) (**E**). Colo205 cells were treated with 1 µM GnRH-AIF or PBS and incubated for 24 h, and DNA was then extracted. A total of 10 µg of DNA of each sample was loaded on a 1.5% agarose gel (**F**). 1: 2.5 µg of 1 kb DNA ladder; 2: 2.5 µg of 1 kb DNA ladder + GnRH-AIF (1 µM); 3: 2.5 µg of 1 kb DNA ladder + GnRH-AIF (1 µM). Cas3 = Caspase-3; PD = Plasmid only. The original Western blot figures can be found in Supplementary File S1.

Our main aim was to design a chimeric protein that induces caspase-independent cell death as a new approach to cancer treatment. Therefore, we tested whether caspase 3 is involved in death caused by the GnRH-AIF chimeric protein. First, we checked caspase 3 activation using Western blot analysis. We incubated Colo205 cells with the GnRH-AIF (1 µM) or GnRH-Caspase-3 (7 µM) chimeric proteins as a positive control. Cells lysates were prepared and analyzed by Western blot using anti-cleaved caspase 3 antibodies. We detected a clear band corresponding to cleaved caspase 3 in the cytoplasm of treated cells with GnRH-Caspase-3 and not with GnRH-AIF (Figure 7A), strongly suggesting that, while GnRH-Caspase-3 caused apoptosis via a caspase-dependent pathway, GnRH-AIF caused death that is caspase-independent, at least in its initial phase.

Next, we tested Caspase 3 activity in vitro, with or without the addition of a pan-caspase inhibitor (zVAD). Colo205 cells were cultured in medium supplemented with or without 20 µM zVAD-FMK for 4 h and then treated with GnRH-AIF, GnRH-Caspase-3, or PBS for 24 and 48 h. There was no evidence of caspase 3 activation following treatment with GnRH-AIF after 48 h (Figure 7B). However, there was a significant increase in caspase 3 activity (27-fold) in cells treated with the GnRH-Caspase-3 chimeric protein, the positive control, compared to the control untreated cells. This increase in caspase 3 activity was completely inhibited in the presence of zVAD. These results affirm that the cell death induced by GnRH-AIF chimeric protein was caused by caspase-independent apoptotic pathways. In parallel, we tested the ability of GnRH-AIF to induce cell death in cancer cells in the presence or absence of zVAD. As can be seen in Figure 7C, treatment of Colo205 and LNCaP target cells with GnRH-AIF with or without the addition of the zVAD inhibitor caused the same levels of cell death, again confirming that death caused by the GnRH-AIF was caspase-independent. Furthermore, while cell death induced by the GnRH-Caspase-3 chimeric protein was similar to that of GnRH-AIF (though at a much higher concentration, see Figure 3A), this was significantly reduced, by ~50%, in the presence of zVAD (Figure 7C).

### 3.10. GnRH-AIF Induces Apoptotic DNA Fragmentation Within Human Target Cancer Cells

The interaction between AIF and DNA is critical for its apoptotic activity. To test whether the GnRH-AIF chimeric protein can bind DNA in vitro, gel retardation experiments were conducted. Various quantities of the GnRH-AIF chimeric protein were incubated with 3 μg of DNA fragments (1 kb DNA ladder) and the electrophoretic mobility of the protein–DNA complexes was analyzed in an agarose gel. In the absence of the GnRH-AIF chimeric protein, the DNA fragments exhibited the anticipated distribution pattern (Figure 7D). However, the addition of the chimeric protein slowed down the migration of DNA fragments, in a concentration-dependent manner. These results demonstrate the ability of GnRH-AIF chimeric protein to bind DNA, at least in vitro. Our findings suggest that the interaction between GnRH-AIF chimeric protein and DNA is critical for inducing DNA fragmentation within target cells.

Since it has been reported previously [35,36] that AIF does not have nuclease activity and may recruit nuclease after it binds to DNA, we assessed the nuclease activity of GnRH-AIF in vitro. Plasmid DNA (Figure 7E) or human genomic DNA (supplementary Figure S1) was incubated with GnRH-AIF, GnRH-AIFinact, or GnRH-Caspase-3 for 20 min at 37 °C. As can be seen in Figure 7E and Figure S1, GnRH-AIF did not cause plasmid and genomic DNA cleavage contrary to the DNase treatment that was used as a positive control. However, incubation of the plasmid with GnRH-AIF changed its running profile, which now appeared as two major bands. We hypothesize that the larger band corresponds to the plasmid after binding with GnRH-AIF chimeric proteins, which causes it to migrate more slowly on the gel. The second band probably represents a change in the conformation of the plasmid caused by the chimeric protein’s binding. Moreover, the GnRH-AIFinact control chimeric protein did not affect the plasmid’s running profile and did not seem to bind to DNA. This is in line with the literature since these mutations were reported to abolish the ability of AIF to bind to the DNA [25]. In addition, we used GnRH-Caspase-3 as a negative control, as this chimera has no ability to bind DNA, thus confirming that it was not the addition of a protein that affected the plasmids’ running profile on the gel. These results are consistent with the findings of our gel retardation experiments and previous research indicating that AIF in the form of the GnRH-AIF chimeric protein has the ability to bind DNA but does not possess nuclease [35,36].

AIF is known to initiate chromatin condensation and DNA fragmentation, leading to caspase-independent apoptotic cell death [21]. Therefore, we tested the ability of the GnRH-AIF chimeric proteins to enter the nucleus and cause DNA fragmentation. Colo205 cells were treated with 1 µM GnRH-AIF chimeric protein for 24 h. Colo205 cells treated with the GnRH-AIF chimeric proteins exhibit classic DNA laddering patterns (Figure 7F), indicating the ability of GnRH-AIF to translocate into the nucleus and to induce apoptotic DNA fragmentation, as expected.

### 3.11. Mechanism of Action of GnRH-AIF Chimeric Protein Within Target Cells

Having demonstrated that the GnRH-AIF chimeric protein was able to enter target cancer cells overexpressing GnRH-R and caused caspase-independent apoptotic cell death, we investigated its mechanism of action upon cell entry. Since it lacks nuclease activity (Figure 7E and Figure S1), AIF has been proposed to directly interact with DNA and to recruit proteins and nucleases to form DNA-degrading complexes [36]. This include PPIA (cyclophilin A) and endonuclease G (ENDOG) [35,36,37,38,39], as well as additional proteins, that form a DNA–degradome complex with AIF to induce DNA fragmentation.

**Figure 8:**
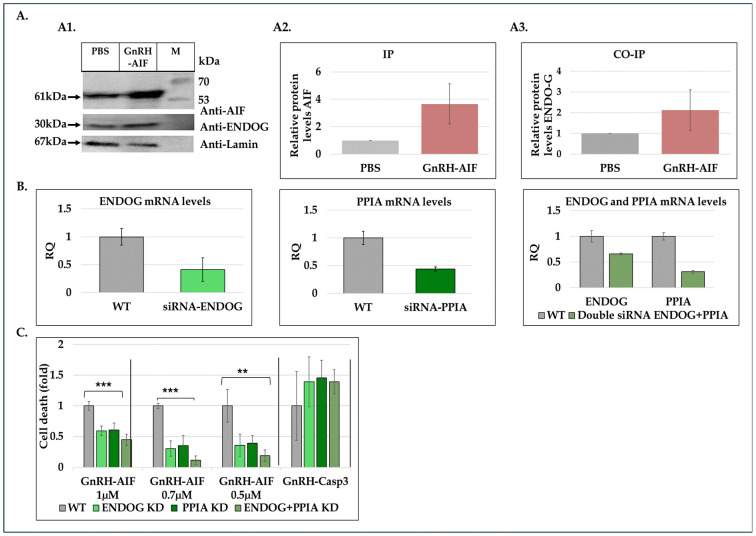
Mechanism of action of GnRH-AIF chimeric protein within target cancer cells. (**A**) Colo205 cells were treated with GnRH-AIF (1 µM) or PBS (control) for 24 h, followed by immunoprecipitation with anti-AIF antibodies and immunodetection of AIF or ENDOG by Western blot analyses (**A1**). Western blots were quantified (**A2**,**A3**). Human mRNA ENDOG or human mRNA PPIA were knocked down by treatment with siRNA-ENDOG or siRNA-PPIA for 48 h in LNCaP cells. (**B**) Cells with or without siRNA treatment were incubated with various concentration of GnRH-AIF, GnRH-Caspase-3, or PBS for 24 h. Results are shown as a fold increase in cell death in the treated cells compared to untreated cells (PBS only). Results are the average of 3–6 repeats ± SD. (**C**) *p* value ≤ 0.01 = **; *p* value ≤ 0.001 = ***. Cas3 = Caspase-3. The original Western blot figures can be found in Supplementary File S1.

First, we investigated the physical interaction between GnRH-AIF and ENDOG by co-immunoprecipitation and Western blot analyses. Cells were treated with GnRH-AIF chimeric proteins or PBS; nuclear fractions were isolated and immunoprecipitated with anti-AIF antibodies. The amount of eluted proteins was 4.5-fold greater in the GnRH-AIF-treated nuclear fraction compared to PBS (Figure 8(A1,A2)). The AIF/ENDOG interaction was then determined by co-immunoprecipitation assays. As can be seen in Figure 8A, ENDOG levels increased by 1.6-fold (Figure 8(A3)) in cells treated with the chimeric proteins compared to control. These findings suggest that the GnRH-AIF entered the cells’ nucleus, as confirmed by the elevated levels of AIF proteins after treatment. Furthermore, the co-immunoprecipitation results suggest that AIF interacts with ENDOG within the nucleus of treated, target cancer cells. To determine whether this interaction is relevant to the apoptogenic action of GnRH-AIF chimeric proteins, we inserted siRNA for ENDOG or PPIA or both simultaneously (Figure 8B) into target cells and tested the ability of GnRH-AIF to cause cell death (Figure 8C). There was a ~40% less cell death in cells treated with the GnRH-AIF chimeric protein after knockdown of ENDOG or PPIA compared to control cells treated with the chimeric protein (Figure 8C). In addition, we observed that silencing both ENDOG and PPIA genes leads to a more significant decrease (80% decrease) in cell death due to GnRH-AIF compared to silencing either one of the genes alone. Treatment with the GnRH-Caspase-3 chimeric protein, a control chimeric protein with no direct relevance to DNA-degrading complexes, was not affected by down-regulating the expression of ENDOG or PPIA or both (Figure 8C). In summary, silencing ENDOG and/or PPIA genes decreased cell death mediated by GnRH-AIF presumably by inhibiting key steps in the apoptotic pathway, such as DNA fragmentation and chromatin condensation.

### 3.12. Anti-Tumor Efficacy of GnRH-AIF Chimeric Proteins in Colon Cancer Human Organoid Models

Finally, we used ex vivo models including human normal colon and CRC organoids to evaluate the potential therapeutic effect of GnRH-AIF chimeric proteins on colon cancer. Cultures of human normal colon and CRC organoids derived from patient samples were established. These organoids recapitulate the characteristics of normal and cancerous colon tissue. CRC and normal organoids were treated with various concentrations of GnRH-AIF or PBS as control for three days.

**Figure 9:**
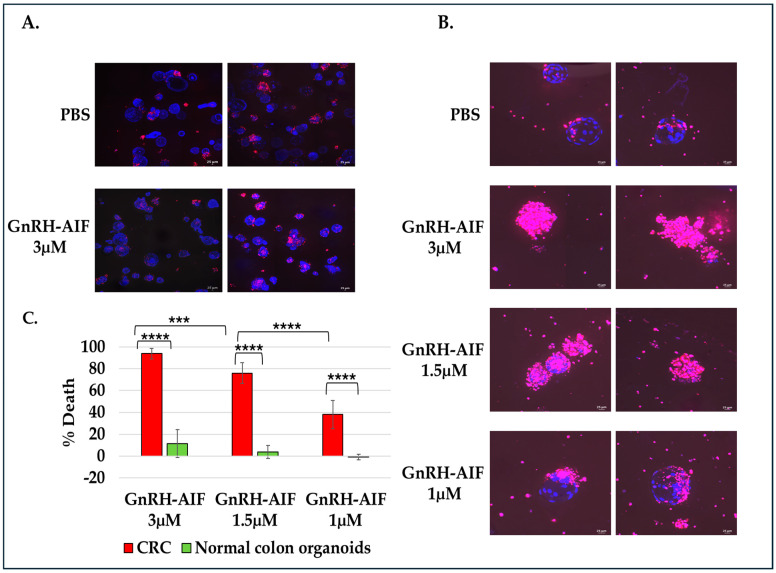
Anti-tumor effect of GnRH-AIF chimeric protein on human colon cancer organoid models. CRC and normal colon organoids were treated with various concentrations of GnRH-AIF (1, 1.5, or 3 µM) or PBS (control) for three days. To measure cytotoxicity, the organoids were stained with Hoechst 33342 and PI to evaluate the total number of cells and dead cells, respectively. Images were taken by confocal microscopy (spinning disk) (magnification 20×). Images of normal colon organoids treated for 72 h with GnRH-AIF (3 µM) or PBS (control) (**A**). Images of CRC organoids treated for 72 h with various concentrations of GnRH-AIF chimeric proteins or PBS (control) (**B**). For each sample/treatment, two images are shown. Quantitation of organoid death calculated for treated organoids as compared to control (PBS) at 72 h after start of treatment (**C**). Results are the average of 3–6 repeats ± SD.; *p* value ≤ 0.001 = ***; *p* value ≤ 0.0001 = ****.

To measure cytotoxicity, the organoids were stained with Hoechst 33342 and PI to evaluate the total number of cells and the number of dead cells, respectively. Treatment with GnRH-AIF chimeric proteins reduced the viability of CRC organoids in a dose-dependent manner, reaching an 80% reduction in the organoids’ viability at the highest concentration tested (Figure 9B,C). Moreover, treatment with GnRH-AIF chimeric protein (3 µM) had no significant effect on the viability of normal human colon organoids (Figure 9A–C), indicating the effect of the newly designed chimeric proteins is specific. Our results demonstrate that GnRH-AIF chimeric proteins are a potent and specific modality for targeted treatment of solid cancers that overexpress the GnRH-R, such as colon cancer, via a caspase-independent mechanism.

## 4. Discussion

Most anticancer treatments trigger tumor cell death through apoptosis, for which initiation of proteolytic action of caspases is a requirement. However, cancerous cells are known to highly regulate the apoptotic pathways, and numerous mutations in various types of cancer cells have been reported to be implicated in chemoresistance and treatment outcome [3,4,5,6,7,8]. Therefore, in this study we aimed to construct a new chimeric cytotoxic protein using AIF in its cleaved apoptotic form as the killing moiety, to induce caspase-independent cell death. The GnRH/GnRH-R system was used to direct the AIF protein into solid cancer cells overexpressing the GnRH-R [19,20,33].

In this study, we constructed, expressed, and highly purified GnRH-AIF chimeric proteins (Figure 1D–F). We demonstrated that GnRH-AIF chimeric proteins enter and kill specifically and very efficiently human cancer cell lines overexpressing GnRH-R (Figure 2 and Figure 4). In addition, we successfully demonstrated that the delivery of AIF via the GnRH targeting system into cancer cells induces a specific caspase-independent apoptotic death (Figure 6 and Figure 7). Furthermore, we produced a clone of GnRH-AIF with an inactive killing component [25], thus confirming that death of the target cells was indeed caused by the killing component of the chimeric proteins (Figure 4B–D). We also designed and produced two additional variants of the chimeric protein, the first including the amino acids 353–613 of AIF corresponding to its C-terminal domain (GnRH-AIFct) and the second variant corresponding to the N-terminal region of AIF (GnRH-AIFnt). Our results demonstrated that the pro-apoptotic activity of AIF resides in its C-terminal domain and that the first N-terminal 352 amino acids of AIF are, most probably, not required for its killing activity (Figure 5E,F). As AIF lacks nuclease activity, it is proposed to directly interact with DNA and disrupt chromatin structure by displacing chromatin-associated proteins and by recruiting nucleases to form DNA-degrading complexes. To demonstrate that these interactions are relevant to the apoptogenic action of AIF, we knocked down relevant partners, PPIA/Endo G, two of those nuclear proteins known to be part of the DNA-degrading complexes within target cells, and tested the ability of GnRH-AIF chimeric protein-treated cells to cause cell death (Figure 8C). Finally, we tested the effect of GnRH-AIF chimeric proteins ex vivo in a human colon cancer organoid model and demonstrated very promising anti-tumor efficacy (Figure 9). Our results established that caspase-independent pathways can be used for cause-specific cell death and suppress tumors as a new approach to treat cancer, in particular solid tumors.

The chimeric protein GnRH-AIF was expressed in a bacterial expression system and aggregated in the IB. Therefore, we had to develop a purification protocol including denaturation of the IB, followed by two steps of purification. In addition, a very sensitive refolding of the protein was needed to produce a highly purified and biologically active chimeric protein. GnRH-AIF chimeric proteins specifically targeted GnRH-R overexpressing cells and induced cells’ specific apoptosis in a dose- and time-dependent manner (Figure 4 and Figure 6). The specificity of the chimeric protein was demonstrated by distinguishing between cancer cells lines expressing and not expressing the GnRH-R (Figure 4). In addition, we found that manipulating the AIF-killing moiety by two point mutations, forming its inactive variant [25], resulted in no killing activity in the targeted cells (Figure 4). Cell cycle analysis and real-time PCR for apoptotic-related proteins indicated that the GnRH-AIF chimeric proteins caused apoptotic cell death (Figure 6). However, measuring caspase 3 activity demonstrated caspase-independent apoptotic cell death, as was our aim (Figure 7A,B). To support our results, another chimeric protein, GnRH-Caspase-3, known to cause caspase-dependent apoptosis, was designed and purified under the same protocol as the GnRH-AIF. Western blot analysis of the cleaved caspase 3 and measurement of caspase 3 activity in vitro demonstrated that GnRH-AIF caused caspase-independent death in its initial phase, unlike GnRH-Caspase-3 which used caspase-dependent apoptotic pathways (Figure 7A,B). In addition, we tested the ability of GnRH-AIF to induce apoptotic cell death in cancer cells under the inhibition of caspase activity (by zVAD treatment). Contrary to GnRH-Caspase-3, the cell death of GnRH-AIF-treated target cells was not affected by zVAD treatment (Figure 7C), confirming that the GnRH-AIF chimeric proteins utilize a caspase-independent pathway to induce cell death within target cells.

Our results demonstrate that GnRH-AIF death is more rapid and effective compared to GnRH-Caspase-3 death (Figure 3), possibly through the activation of a direct apoptotic pathway. On the other hand, GnRH-Caspase-3 may trigger cell death through a different mechanism, likely involving the activation of caspase 3, which might be relatively slower or less effective. Tumor cells lacking a functional caspase-dependent pathway can resist conventional chemotherapeutic agents or radiation, which often work by triggering caspase-dependent apoptosis pathways [40]. Research is ongoing to develop treatments that can bypass or target alternative cell death pathways [41]. Therefore, our study illustrates the promising potential of GnRH-AIF chimeric protein treatment to overcome the problems encountered with defects in caspase-dependent apoptotic pathways and moreover to be effective for a large number of solid cancers overexpressing GnRH-R.

In order to get a deep understanding of AIF’s mechanism of action while inducing cell death, we investigated how AIF, lacking nuclease activity itself, contributes to DNA degradation [36]. Previous research demonstrated the implication of several proteins, such as PPIA and ENDOG, in the apoptotic role of AIF [35,38,42]. Immunoprecipitation and co-immunoprecipitation experiments using nuclear extracts from treated and untreated cells demonstrated the physical interaction between GnRH-AIF and Endonuclease G (Figure 8A). By utilizing siRNAs targeting ENDOG and PPIA proteins, we also demonstrated that their knockdown significantly reduced cell death induced by the GnRH-AIF chimeric protein (Figure 8C). One possible explanation is that both ENDOG and PPIA recruitment to the AIF facilitates DNA degradation. Silencing ENDOG and PPIA genes could abrogate these interactions, thereby inhibiting the AIF downstream effects, leading to decreased cell death. This interpretation implies a dependency of GnRH-AIF-mediated cell death on the presence and activity of ENDOG and PPIA. Further investigations are needed to fully understand the precise mechanism by which AIF forms a complex with ENDOG and PPIA and probably other undescribed proteins to perform its apoptotic role. In addition, it is critical to understand the role of each protein within the complex known as the degradome complex [35,38,42].

We also tested the nuclease activity of AIF in vitro, as it was recently reported that AIF has the ability to act as a nuclease in certain conditions and without the assistance of any proteins [42]. However, our results showed that AIF, in the form of a chimeric protein, only has the ability to bind; however, it does not bind to cleaved genomic DNA or plasmid DNA in vitro (Figure 7E). This article [42] contradicts our current findings, which point out the involvement of ENDOG and PPIA in the mechanism by which the GnRH-AIF chimeric protein induces apoptosis in target cells. Further investigation is needed to clarify the detailed molecular mechanism of AIF protein.

The AIF protein is composed of three structural domains: the FAD-binding domain, the NADH-binding domain (oxidoreductase part of AIF), and the C-terminal domain (apoptotic function). Previous studies demonstrated that the C-terminal domain of AIF is necessary and sufficient to induce chromatin condensation and large-scale DNA fragmentation leading to caspase-independent apoptosis [34]. Therefore, we constructed additional GnRH-AIF chimeric proteins variants. The GnRH-AIFct chimeric protein, including the only AIF C-terminal domain, was indeed able to cause cell death, however, less effectively than the original GnRH-AIF, full-length, chimeric protein. In contrast, the GnRH-AIFnt chimeric protein, containing the first 352 amino acids of the N-terminal domain, did not kill the targeted cells (Figure 5E,F). These results confirmed that the C-terminal domain is the apoptotic domain of the AIF protein and confirmed that the AIF component of the chimeric proteins is the killing moiety responsible for the killing activity of the chimeric proteins. Further co-immunoprecipitation experiments should be performed to determine the exact region in the C-terminal domain by which AIF binds to the DNA.

Most importantly, the activity of GnRH-AIF was remarkable on primary organoid cultures from human colorectal cancer, and the specificity was proven using organoids originating from normal colon culture. In recent years, tumor organoid models have been widely used for cancer drug testing [43]. Produced by using stem cell-derived tissues and cultivated under specific conditions, organoids mimic the heterogeneity and the complexity of the original tumor, therefore representing a promising tool for drug testing for human cancer therapies [44]. This result further demonstrates the potential of the GnRH-AIF chimeric protein as a new highly effective modality for the treatment of solid tumors (Figure 9).

In this work, we showed further proof of the efficacity of the GnRH peptide as a targeting moiety for GnRH-R overexpressing cells. We used the GnRH/GnRH-receptor system to direct the AIF into cancer cells expressing the receptor [19,20]. Previously, using the bacterial toxin *Pseudomonas exotoxin A* (PE) as well as human apoptotic proteins, such as Bax, Bak, Bik, and DFF40 [16,19,20], we constructed a number chimeric proteins based on GnRH as the “targeting” component. Testing these GnRH-based chimeric proteins on different cancer cell lines, primary cultures, as well as in colon cancer models in mice [13,45], we were the first to report that a wide variety of solid tumors that are not part of the reproductive system, such as colon cancer, kidney cancer, and many other solid tumors, overexpress the GnRH-R/binding sites [16,28,33].

AIF is involved in other cellular contexts such as parthanatos [46], a form of programmed cell death linked to neurodegenerative diseases such as Alzheimer’s and Parkinson’s. Parthanatos involves a cascade of signaling steps that cause cell death, induced by PARP-1 overactivation, and involves AIF and a macrophage migration inhibitory factor (MIF) [47]. Therefore, manipulating AIF activity opens up new opportunities for developing therapeutic strategies for treating various diseases such as Alzheimer’s and Parkinson’s [48].

**Figure 10:**
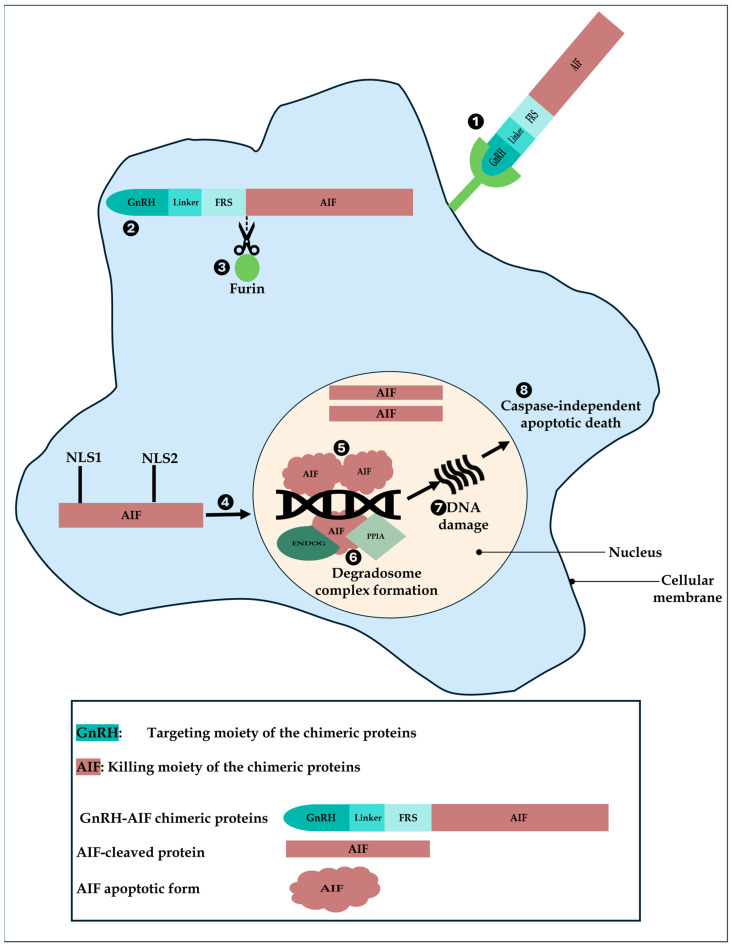
Proposed mechanism of action of GnRH-AIF chimeric proteins. Binding of the targeting chimeric protein moiety GnRH to the GnRH-receptor overexpressed in cancer cells (1). Internalisation of GnRH-AIF in target cells (2). Cleavage of the chimeric protein at the Furin site by Furin enzyme inducing the separation of the AIF moiety from the targeting motif (3). Translocation of the AIF-cleaved proteins to the nucleus (4). Binding of AIF soluble form to the DNA strands (5). Recruitment of ENDOG and PPIA to the DNA by AIF, leading to the formation of the degradasome complex (6). Large-scale chromatin condensation and DNA fragmentation (7). Activation of the caspase-independent apoptotic pathways leading to cell death (8). FRS = Furin site.

Based on our results, we propose a model of action of GnRH-AIF in GnRH-R overexpressing cancer cells (Figure 10). First, GnRH-AIF enters the cells and localizes primarily in the cytosol where it is cleaved at the Furin site. The AIF-cleaved apoptotic protein translocates into the nucleus, binds to DNA, recruits ENDOG and PPIA among other unknown proteins, causing DNA fragmentation, and finally leads to caspase-independent apoptotic death.

GnRH-AIF is a new chimeric protein designed to bypass the classical caspase-dependent apoptotic pathways. The highly encouraging results described here regarding GnRH-AIF chimeric protein’s killing specificity and efficacy in vitro against human target cancer cells lines and ex vitro in human colon cancer organoid models suggest it as a novel treatment for solid cancers overexpressing the GnRH-R. Solid tumors account for a large and diverse group of cancers that can form metastases, are hard to treat, and have a high rate of mortality [49]. For example, colorectal cancer was the fourth-leading cause of cancer death in both men and women younger than 50 years in the late 1990s but is now first in men and second in women [50]. In addition, we suggest that AIF in the form of chimeric proteins can be applied to other types of tumors, using the appropriate targeting moiety so expanding therapy based on AIF proteins to a variety of other tumors.

## 5. Conclusions

In this study, we developed and tested a novel, engineered protein molecule that induces caspase-independent apoptotic death for targeted cancer treatment of solid tumors, the GnRH-AIF chimeric protein. Our results suggest that AIF in the form of chimeric proteins can be applied to other types of tumors, using the appropriate targeting moiety, so expanding targeted therapy based on AIF proteins to a variety of other tumors. In addition, being such a potent apoptotic inducer, cleaved AIF can be considered a very promising target for developing additional cancer therapies, based on its specific manipulation within cancer cells. Most importantly, our promising results established that caspase-independent pathways can be utilized for cause-specific cell death and suppress tumors’ growth as an innovative approach to treat cancer.

## Figures and Tables

**Table 1:** Primers used for PCR Reactions.

GnRH-AIF Chimeric Protein Variants	Sense/ Antisense	PCR Reaction 1	PCR Reaction 1
GnRH-AIFinact	sense	GTTTGCCCACAGTTGGTGTTTTTGCAGCAGCAACTCACAAGACAACCCCGCATCTGCCACAGAGCAGTCAGG ACTG	CCTGAGCGAGACGAAATACGCGATC
	antisense	GATCGCGTATTTCGTCTCGCTCAGG	CAGTTCCTGACTGCTCTGTGGCAGATGCGGGGTTGTCTTGTGCAGTTGCTGCTGCAAAAACACCAACTGTGGGCAAAC
GnRH-AIFct	sense	CCTGAGCGAGACGAAATACGCGATC	AGAATTCTCTCCACGGCGGGCACGTATGGAAAAAGTCAGACGAGAG
	antisense	CCCCCTCTCGTCTGACTTTTTCCATACGTGCCCGCCGTGGAGAGAATTC	GATCGCGTATTTCGTCTCGCTCAGG
GnRH-AIFnt	sense	CGATATAAAGTTGGGAAGGAGGCGGTGAGAGCTCATTATTTGTGG	CCTGAGCGAGACGAAATACGCGATC
	antisense	GATCGCGTATTTCGTCTCGCTCAGG	CAAGTCCACAAATAATGAGCTCTCACCGCCTCCTTCCCAACTTTATATC

**Table 2:** Primers used for real-time PCR reactions.

Primer Name	Sense/Antisense	Primer Sequence
hENDOG (exons 2–3 134 bp)	Sense	GCAGCTACCAAAACGTCTATGT
	Antisense	CACCTTGAAGAAGTGTGTGGG
hPPIA (exons 2–3, 275 bp)	Sense	CCCACCGTGTTCTTCGACATT
	Antisense	GGACCCGTATGCTTTAGGATGA
hBax (exons 4–5, 116 bp)	Sense	TCTGACGGCAACTTCAACTG
	Antisense	CAGCCCATGATGGTTCTGA
hBcl2 (exons 2–3, 134 bp)	Sense	CCCCTGGTGGACAACATC
	Antisense	GGCCGTACAGTTCCCAAA
hG6PD (exons 6–7, 283 bp)	Sense	TCTACCGCATCGACCACTACC
	Antisense	GCGATGTTGTCCCGGTTC

“h” indicates human genes.

## Data Availability

Data are contained within the article and Supplementary Materials.

## Supplementary Materials

The following supporting information can be downloaded at: https://www.mdpi.com/article/10.3390/cancers17071179/s1, Figure S1: AIF protein, in the form of GnRH-AIF chimeric protein, has no nuclease activity; Figure S2: Knockdown of ENDOG confirmed at the protein level; Table S1: GnRH-R expression (%) on human cancer cell lines; File S1: The original Western blot figures.
