# Supplementary material for: Inducing Targeted, Caspase-Independent Apoptosis with New Chimeric Proteins for Treatment of Solid Cancers
Source: Cancers (Basel). 2025 Mar 31;17(7):1179. doi: 10.3390/cancers17071179 (PMC11988119; doi:10.3390/cancers17071179)
Supplement: Supplementary file 1 [file cancers-17-01179-s001.zip › cancers-3486175-supplementary.pdf]

Supplementary Materials

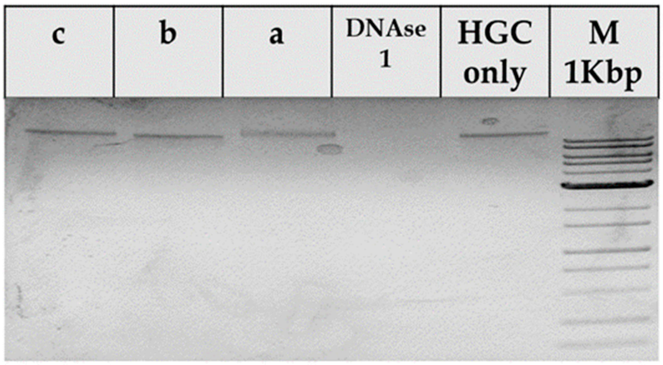

Figure S1: AIF protein, in the form of GnRH-AIF chimeric protein, has no nuclease activity. Human Genomic DNA (500 ng) was mixed and incubated with GnRH-AIF (12 mg) (a), GnRH-AIFinact (b) or GnRH-Caspase3 (c) or 1 IU of DNAse for 1 min (DNAse 1) and incubated for 20 min at 37°C and analyzed by agarose gel electrophoresis (0.8%). HGD=Human genomic DNA.

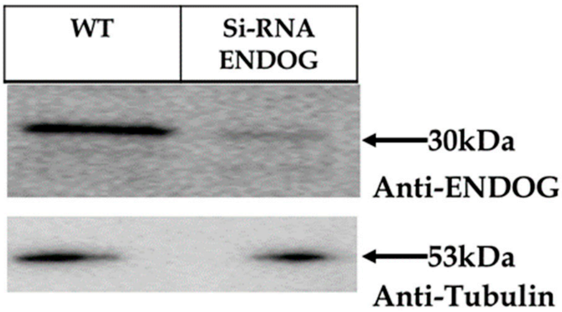

Figure S2: Knockdown of ENDOG confirmed at the protein level. Knockdown of human ENDOG following treatment with siRNA-ENDOG for 48hrs in LNCaP cells was confirmed by western blot analysis using anti-ENDOG, and anti-Tubulin as a loading control.

Table S1: GnRH-R expression (%) on human cancer cell lines.

Cell line	Cell Type	% Expression GnRH-R
Colo205	Colon adenocarcinoma	72
LNCaP	Prostate adenocarcinoma	70
HepG2	Hepatocarcinoma	56
SW48	Colon adenocarcinoma	68
MCF-7	Breast adenocarcinoma	51
HEK-293	Renal adenocarcinoma	58
T24P	Urinary bladder carcinoma	6
A204	Rhabdomyosarcoma	5

Adhered cells were collected by scraping with a rubber policeman and incubated at 37°C for 30 min until recovery. The cells were pelleted and resuspended in binding buffer (PBS containing 3% fetal calf serum and 0.2% sodium azide). For each cell line, three eppendorf tubes (each containing 10^6 cells per 400μl) were used. In the first tube, the cells were incubated with 10μg of the GnRH-Caspase3. In the second tube, the cells were incubated with binding buffer only. To address specificity of binding through the GnRH moiety. 10μg of a control chimeric protein, based on an irrelevant targeting moiety fused to Caspase3, and expressed in a similar way as GnRH-Caspase3, was added to the cells in the third tube. The tubes were incubated for 90 min in a roller at 4.the cells were then washed three times with the binding buffer and centrifuged (3 min, 950 x g, 4°C). FITC-Anti-Caspase-3 (Santa Cruz, CA, USA) was added to each tube (1 μl in 100 μl of cells in binding buffer) and incubated for 1 h at 4°C. Three washes were performed as described above. Finally, the cells were resuspended in 500 μl of binding buffer and were analyzed by FACScan, using the CELLQuest program (Becton Dickinson, Immunocytomery System, San Jose, CA, USA). % Expression represents the percentage of stained cells [33].
