# Supplementary figures and images for: Inducing Targeted, Caspase-Independent Apoptosis with New Chimeric Proteins for Treatment of Solid Cancers
Source: Cancers (Basel). 2025 Mar 31;17(7):1179. doi: 10.3390/cancers17071179 (PMC11988119; doi:10.3390/cancers17071179)

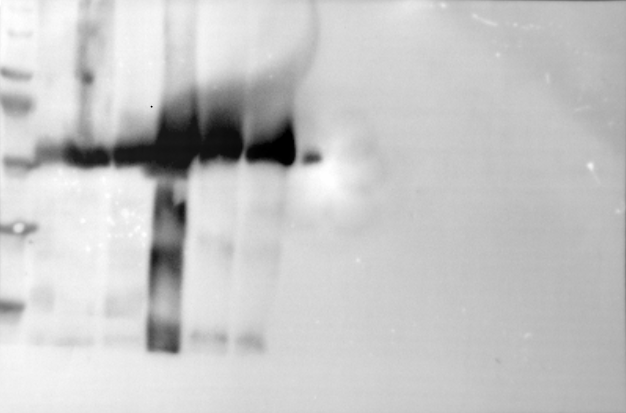

Supplement: Supplementary file 1 [file cancers-17-01179-s001.zip › File S1/fig 1E anti-His.PNG]

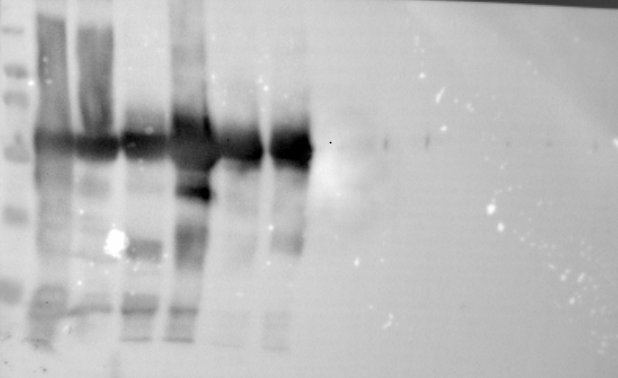

Supplement: Supplementary file 1 [file cancers-17-01179-s001.zip › File S1/fig 1F anti-AIF.PNG]

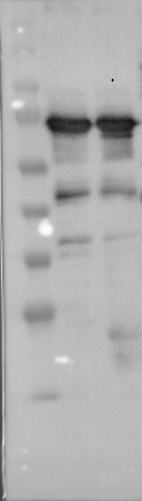

Supplement: Supplementary file 1 [file cancers-17-01179-s001.zip › File S1/fig 1G anti-His.PNG]

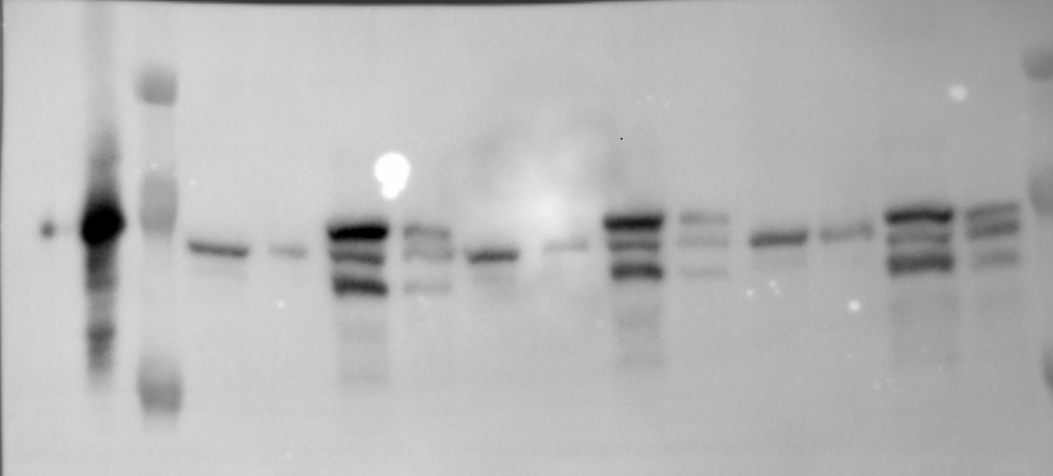

Supplement: Supplementary file 1 [file cancers-17-01179-s001.zip › File S1/Fig 2B anti-AIF.PNG]

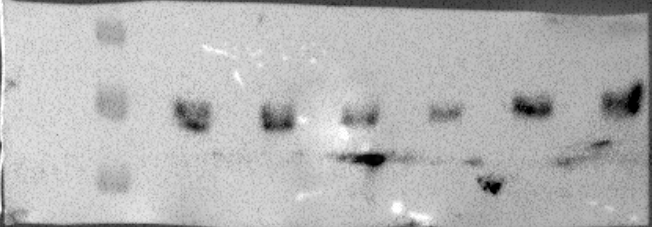

Supplement: Supplementary file 1 [file cancers-17-01179-s001.zip › File S1/Fig 2B anti-SF2.PNG]

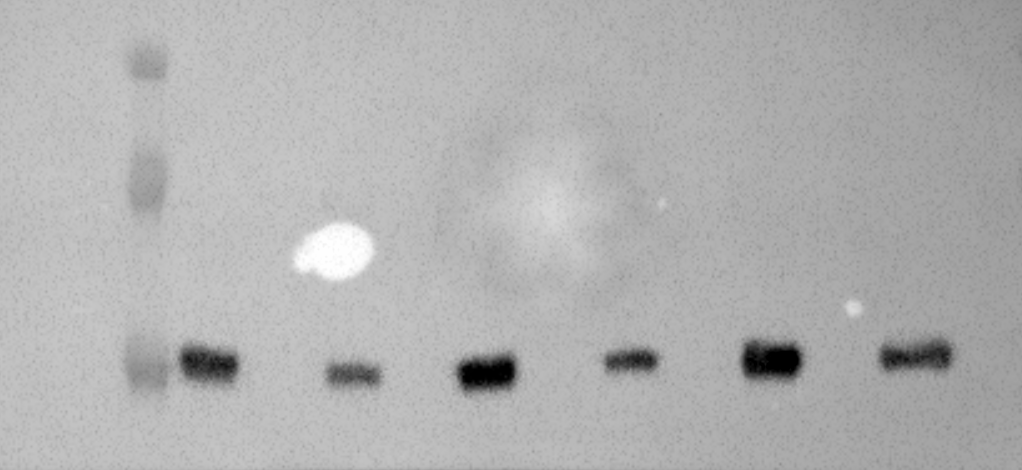

Supplement: Supplementary file 1 [file cancers-17-01179-s001.zip › File S1/Fig 2B anti-Tubulin.PNG]

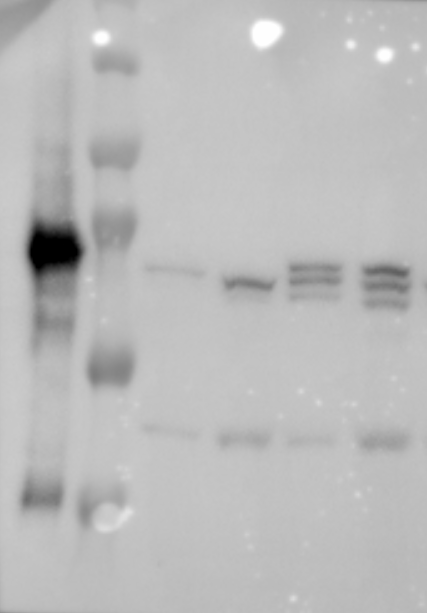

Supplement: Supplementary file 1 [file cancers-17-01179-s001.zip › File S1/Fig 2C anti-AIF.PNG]

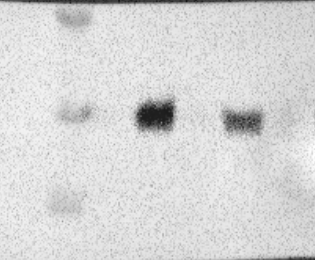

Supplement: Supplementary file 1 [file cancers-17-01179-s001.zip › File S1/Fig 2C anti-SF2.PNG]

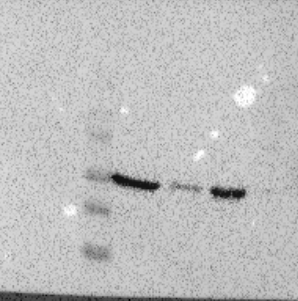

Supplement: Supplementary file 1 [file cancers-17-01179-s001.zip › File S1/Fig 2C anti-Tubulin.PNG]

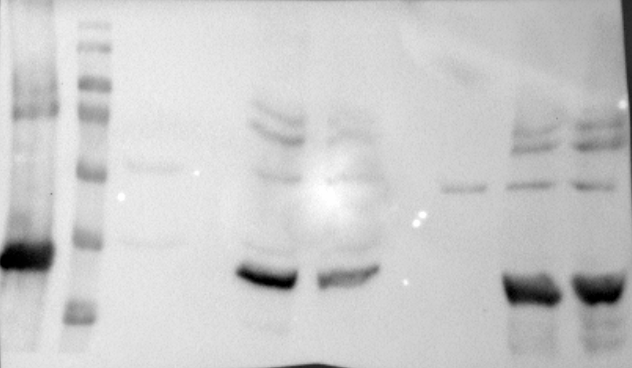

Supplement: Supplementary file 1 [file cancers-17-01179-s001.zip › File S1/Fig 5B anti-AIF.PNG]

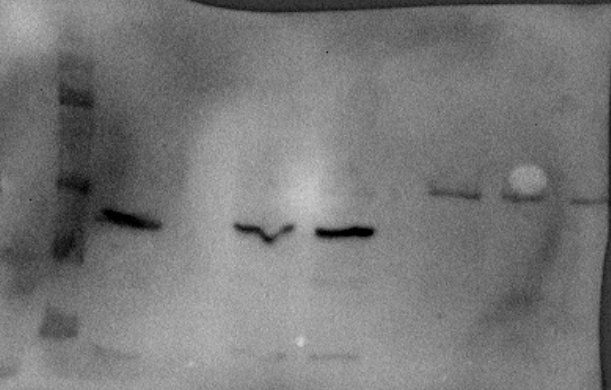

Supplement: Supplementary file 1 [file cancers-17-01179-s001.zip › File S1/Fig 5B anti-Lamin.PNG]

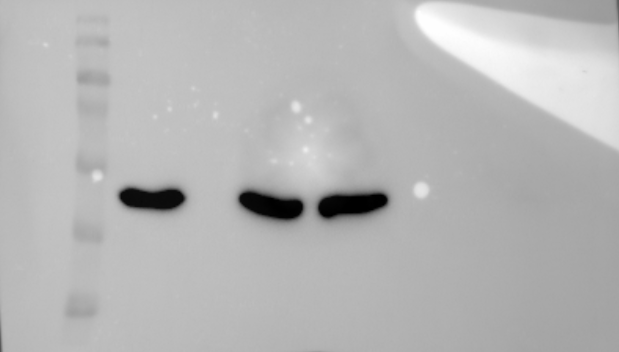

Supplement: Supplementary file 1 [file cancers-17-01179-s001.zip › File S1/Fig 5B anti-Tubulin.PNG]

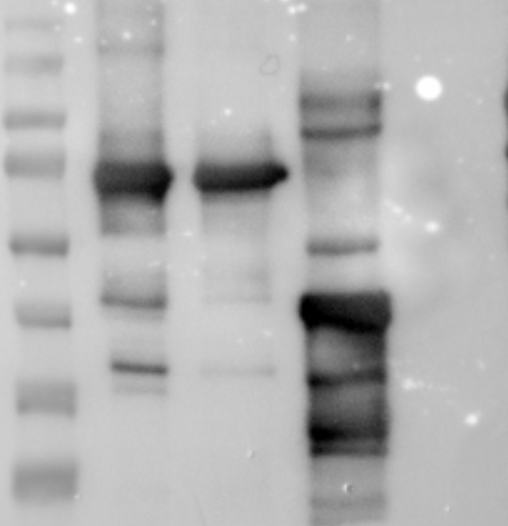

Supplement: Supplementary file 1 [file cancers-17-01179-s001.zip › File S1/fig 5C anti-AIF.PNG]

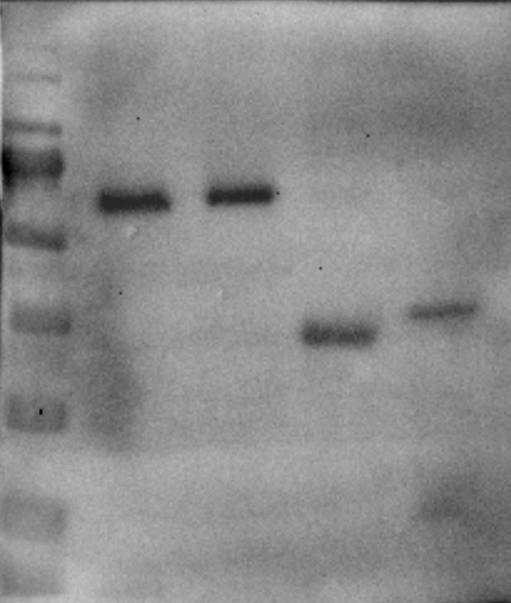

Supplement: Supplementary file 1 [file cancers-17-01179-s001.zip › File S1/fig 5D anti-His.PNG]

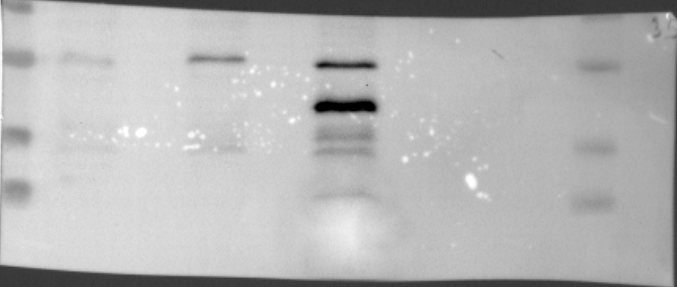

Supplement: Supplementary file 1 [file cancers-17-01179-s001.zip › File S1/Fig 7A anti-Cleaved-Caspase-3.PNG]

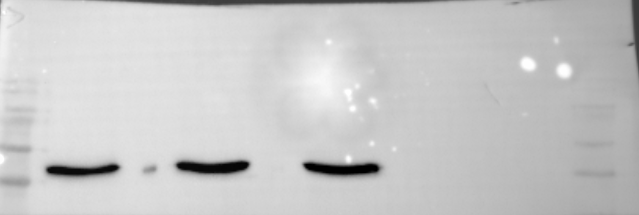

Supplement: Supplementary file 1 [file cancers-17-01179-s001.zip › File S1/Fig 7A anti-Tubulin.PNG]

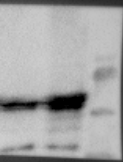

Supplement: Supplementary file 1 [file cancers-17-01179-s001.zip › File S1/Fig 8A1 anti-AIF.PNG]

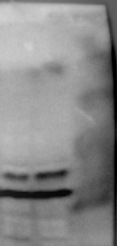

Supplement: Supplementary file 1 [file cancers-17-01179-s001.zip › File S1/Fig 8A1 anti-ENDOG.PNG]

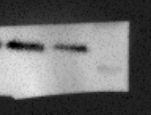

Supplement: Supplementary file 1 [file cancers-17-01179-s001.zip › File S1/Fig 8A1 anti-Lamin.PNG]

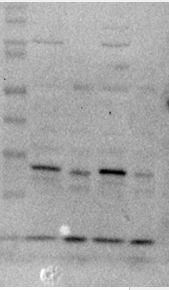

Supplement: Supplementary file 1 [file cancers-17-01179-s001.zip › File S1/Fig S2 anti-ENDOG.PNG]

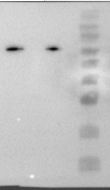

Supplement: Supplementary file 1 [file cancers-17-01179-s001.zip › File S1/Fig S2 anti-Tubulin.PNG]
